# Searching for Hub Genes of Quince–Basil Co-Administration Against Atherosclerosis Using Bioinformatics Analysis and Experimental Validation

**DOI:** 10.3390/ph17111433

**Published:** 2024-10-25

**Authors:** Sendaer Hailati, Meng-Yuan Han, Dilihuma Dilimulati, Nuerbiye Nueraihemaiti, Alhar Baishan, Alifeiye Aikebaier, Wen-Ting Zhou

**Affiliations:** 1 Department of Pharmacology, School of Pharmacy, Xinjiang Medical University, Urumqi 830017, China; sendaer@stu.xjmu.edu.cn (S.H.); hanmengyuan@stu.xjmu.edu.cn (M.-Y.H.); dilihuma@stu.xjmu.edu.cn (D.D.); nurbiye@stu.xjmu.edu.cn (N.N.); alhar@stu.xjmu.edu.cn (A.B.); alfi110@163.com (A.A.); 2 Xinjiang Key Laboratory of Natural Medicines Active Components and Drug Release Technology, Urumqi 830017, China; 3 Xinjiang Key Laboratory of Biopharmaceuticals and Medical Devices, Urumqi 830017, China; 4 Engineering Research Center of Xinjiang and Central Asian Medicine Resources, Ministry of Education, Urumqi 830017, China

**Keywords:** hub genes, quince, basil, atherosclerosis, bioinformatics analysis, molecular docking, molecular dynamics simulation

## Abstract

Background: Atherosclerosis (AS) has one of the highest rates of morbidity and death globally. *Cydonia oblonga* Mill. (quince, COM) and *Ocimum basilicum* L. (basil, OB) are Uyghur medicines that are often used for anti-inflammatory, anti-tumor, and cardiovascular disease treatment. This study aimed to uncover the hub genes of the quince-basil co-administration against AS and validate them. Methods: Network pharmacology analysis and bioinformatics analysis methods were utilized to map the network and obtain four hub genes. Experiments were performed in vivo and in vitro using HUVEC and *zebrafish* to validate the therapeutic effect of COM-OB co-administration against AS. Finally, the hub genes were validated by Western blot. Results: Screening by network pharmacology analysis and bioinformatics analysis obtained a total of 3302 drug targets, 1963 disease targets, and 1630 DEGs. A series of bioinformatic analyses were utilized to ultimately screen four hub genes, and the stability was also verified by molecular docking and molecular dynamics. COM-OB total flavonoids co-administration significantly decreased PA-induced lipid deposition in HUVEC and reduced high cholesterol-induced fat accumulation in *zebrafish*. Western blot results showed that COM-OB co-administration significantly affected the expression of hub genes. Conclusions: The study identified and validated four hub genes, COL1A1, COL3A1, BGLAP, and NOX4, thus providing a rationale for the treatment of AS with COM and OB co-administration.

## 1. Introduction

Atherosclerosis (AS) is the most common underlying lesion of coronary artery disease (CAD), peripheral artery disease (PAD), and cerebrovascular disease [1,2,3]. It is a major cause of mortality in both developed and developing countries, accounting for an estimated 17.9 million deaths each year and as much as 32% of all deaths due to CVDs, according to data provided by the World Health Organization (WHO) (https://www.who.int/health-topics/cardiovascular-diseases#tab=tab_1, accessed on 16 December 2023) [4]. Its most frequent complications include myocardial infarction (MI) and stroke due to spontaneous thrombotic vascular obstruction, both of which are also the most prevalent causes of death globally. The treatment of complications is emphasized by current clinical guidelines [5]. Despite continued advances in the treatment of cardiovascular disease, it remains the leading cause of death in the global population.

There is now growing evidence that immunologic and anti-inflammatory mechanisms play a pivotal role in the development dimension of AS [1,6,7]. The majority of immunocytes differed significantly between the AS group and the control group, such as M0 and M1 macrophages, monocytes, and T cells [8,9]. Inflammation has been proven to influence AS plaque development and evolution. AS is caused by endothelial damage, lipid metabolism abnormalities, and hematological disturbances. It is believed that the atherosclerotic progression is linked to inflammatory changes in endothelial cells that are induced by blood flow [10]. Substantial levels of low-density lipoproteins (LDLs) are transformed into oxidized LDLs (ox-LDLs), and they accumulate on the inner walls of the arteries, which leads to AS plaque creation [11,12]. Cardiovascular disease can be prevented through effective AS prevention and treatment [13]. Currently, lipid-lowering drugs such as statins are the first-line drugs for clinical treatment both domestically and internationally, but increasing evidence of the multiple side effects of statins, rhabdomyolysis, and liver function impairment has been identified, so establishing safer, more effective treatments is of great interest.

AS is an insidious development that drugs or surgery alone cannot reverse. Unfortunately, no molecular biomarkers have yet been fully utilized for directing the treatment of AS in the clinic [14]. Therefore, the search for new and powerful biomarkers is a viable option for improving personalized medicine for AS.

Traditional Chinese medicine (TCM) is a distinctive Chinese healthcare resource that humans have used for over 2000 years [13]. The advantages TCM has in AS treatment cannot be underestimated. With rich targets, remarkable therapeutic effects, and high security, TCM has enormous potency for the alleviation of symptoms and interrupting the progress of the illness [15].

*Cydonia oblonga* Mill. (COM) is a species of plant from the Rosaceae family [16]. It is also known as Kinashi, Soil papaya, and Biye and is a Uyghur folk medicine herb that grows in the Hotan and Kashgar regions of Xinjiang. COM is rich in a variety of active ingredients that have potential curative value. It contains polysaccharides, phenolic acids, flavonoids, alkaloids, organic acids, and volatile oils [17]. Previous studies have demonstrated how COM extracts can be used as an antihypertensive and for anti-inflammatory, hypoglycemic, antioxidant, hypolipidemic, and antithrombotic biological activities [18,19,20,21,22]. These activities contribute significantly to the pathological factors that are involved in the onset and progression of AS [23]. *Ocimum basilicum* L. (OB) is a plant from the Labiatae family that is found in India, Iran, Africa, and the tropical and subtropical regions of Xinjiang, Yunnan, and Guangxi in China [24]. Its leaves also have medicinal properties because they contain polyphenols, phenolic acids, and flavonoids [24]. Domestic and international studies have proven that it can lower blood glucose, reduce inflammation, treat respiratory diseases, reduce cancer and diabetes, and has antioxidant, antibacterial, immunomodulatory, and hepatoprotective properties [25,26,27,28]. It is a conventional therapeutic medication that is used for the treatment and prevention of CAD by the Uyghur people of Xinjiang [29].

The combination of drugs is of major importance in CAD prevention and therapy. Combining two drugs with similar effects and efficacy can effectively prevent and control the development of the disease and improve the therapeutic effect through synergistic effects. The material basis of the effective components of traditional Chinese medicine is clear, and the mechanism is relatively obvious, which gives full play to the therapeutic advantages of the original formula while also eliminating interference by non-pharmacological components, reducing toxic side effects, and overcoming disadvantages relating to the complexity of the traditional components and the difficulty in controlling the quality of the traditional formula. This makes the treatment more targeted with a better safety profile, making it suitable for popularization.

Our previous study found the combination of COM total flavonoids and OB total flavonoids to effectively reduce the levels of vascular damage factors, protect the vascular lining, reduce the occurrence of inflammatory reactions, and prevent AS generation [30,31,32]. Therefore, this study utilizes the active ingredients and targets of COM total flavonoids and OB total flavonoids as a means of predicting the hub genes and potential mechanisms for AS treatment.

## 2. Results

### 2.1. Description of This Study

**Figure 1:**
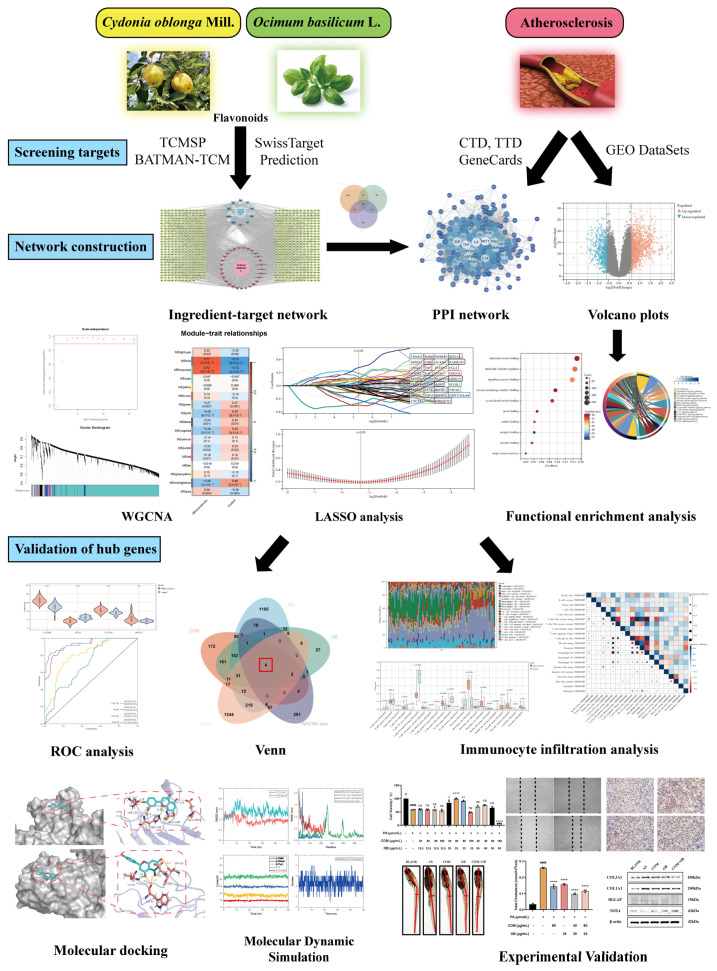
Flowchart of the study of COM and OB co-administration against AS. In this paper, active compounds and targets of *Cydonia oblonga* Mill. and *Ocimum basilicum* L. were collected using TCMSP, BATMAN-TCM, and SwissTarget Prediction databases. CTD, TTD, and GeneCards databases were utilized to collect atherosclerosis targets, and the collected drug and disease targets were intersected and analyzed for PPI network mapping. Meanwhile, the normal and diseased genes of atherosclerosis patients collected through the GEO database were screened for DEGs, volcano and heat maps were drawn, and GO analysis and KEGG pathway analysis were carried out using R. The screened differential genes were subjected to WGCNA. Finally, drug targets, disease targets, DEGs, and module genes were then uploaded to Venn analysis to obtain four hub genes, which were validated by LASSO analysis, ROC analysis, immune infiltration analysis, molecular docking, and molecular dynamics simulation. The therapeutic effects of COM-OB were explored by in vitro and in vivo experiments and the hub genes were validated using Western blot. Note: TCMSP: Traditional Chinese Medicine Systems Pharmacology Database and Analysis Platform; BATMAN-TCM: Bioinformatics Analysis Tool of Molecular Mechanism of Traditional Chinese Medicine; CTD: the Comparative Toxicogenomics Database; TTD: the Therapeutic Target Database; GEO database: Gene Expression Omnibus database; PPI network: the protein–protein interaction network; DEGs: differentially expressed genes; GO analysis: gene ontology; KEGG analysis: Kyoto Encyclopedia of Genes and Genomes; WGCNA analysis: the weighted gene co-expression network analysis; LASSO analysis: least absolute shrinkage and selection operator; ROC analysis: receiver operating characteristic. ns represents statistically insignificant; * represents *p* < 0.05; ** represents *p* < 0.01; **** represents *p* < 0.0001; #### represents *p* < 0.0001.

The main goals of the study were to discover the underlying molecular mechanisms and screen the hub genes of the Uyghur medicine quince and basil, which is co-administered for AS therapy. In Figure 1, the study’s flowchart is displayed. First, COM, OB, and AS targets were obtained using the TCMSP, BATMAN, and GeneCards databases. Then, the ingredient–target network and PPI network were mapped. Using R, DEGs were produced based on 104 samples from the GEO database. To identify the blue module genes most strongly linked to AS, the WGCNA was carried out. A total of 22 immunocytes were then subjected to an immunological infiltration study using the CIBERSORT algorithm. Ultimately, LASSO regression analysis, ROC curve analysis, molecular docking, and molecular dynamics simulations were used to identify and validate four genes as the hub genes. In addition, HUVEC and *zebrafish* were used to perform in vitro and in vivo experiments to validate the therapeutic effects of COM-OB. Finally, the hub genes were validated using Western blot.

### 2.2. COM, OB, and AS Target Collection

The literature and databases screening were used to obtain 10 active ingredients of COM and 34 active ingredients of OB. In all, 810, 521, and 1971 ingredient targets were then obtained from the TCMSP [33], BATMAN-TCM [34], PubChem Database [35], and Swiss-Target Prediction databases [36], respectively (Table 1). At the same time, 506, 35, and 1422 AS-associated targets were obtained from the CTD [37], the TTD [38], and GeneCards [39] databases.

### 2.3. Network Construction

**Figure 2:**
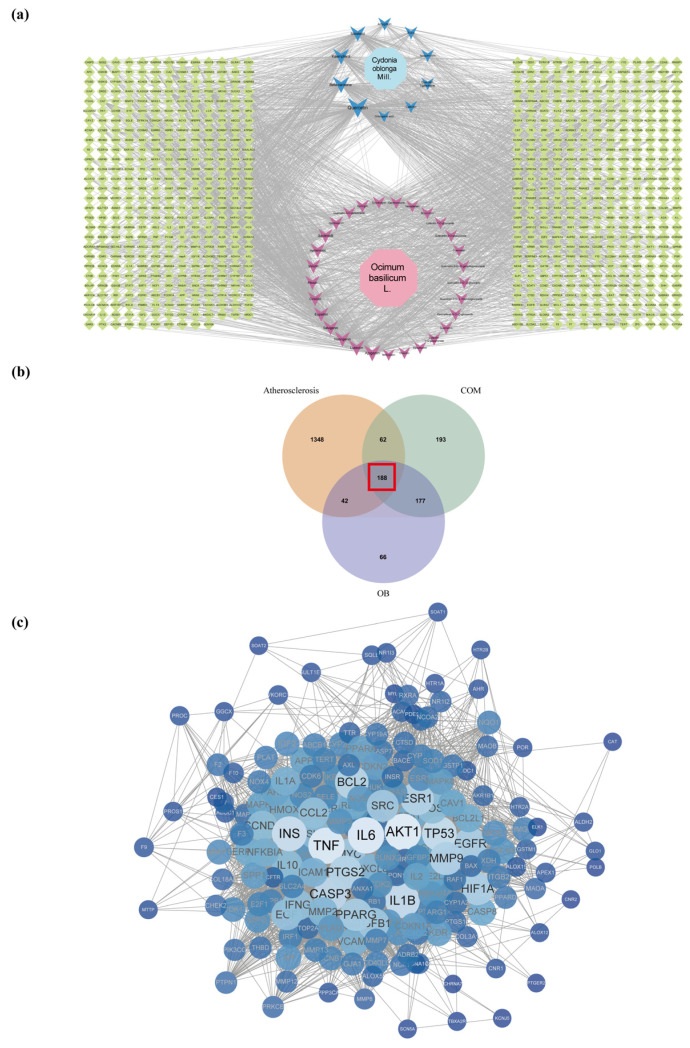
(**a**) Ingredient–target network, ingredient and target sizes are ranked according to the degree values analyzed by Cytoscape, with blue and pink being the active ingredients of COM and OB, and green being the target. (**b**) Venn diagram obtained 188 overlapping targets for drug COM and OB with disease AS. (**c**) PPI network, protein–protein interactions were analyzed for 188 intersecting targets, and proteins were ranked according to Degree values analyzed by Cytoscape.

The active ingredients and targets were exported into Cytoscape 3.8.0 software to create an ingredient–target network (Figure 2a). It included 2 drugs, 44 active ingredients, and 3302 ingredient targets. The ingredients and targets were ordered based on degree value. Higher degree values equate to more involved bio-functions and greater biological importance (Table 2). A total of 188 overlapping genes of COM, OB, and AS were obtained by a Venn analysis of the treatment genes (Figure 2b). The protein interaction information of 188 genes was then obtained from the STRING database [40]. Finally, the interaction file was exported into Cytoscape 3.8.0 to construct the protein–protein interaction (PPI) network (Figure 2c, Table 3). AKT1, IL6, and TNF were the top three in terms of degree value.

### 2.4. Acquisition of DEGs

**Figure 3:**
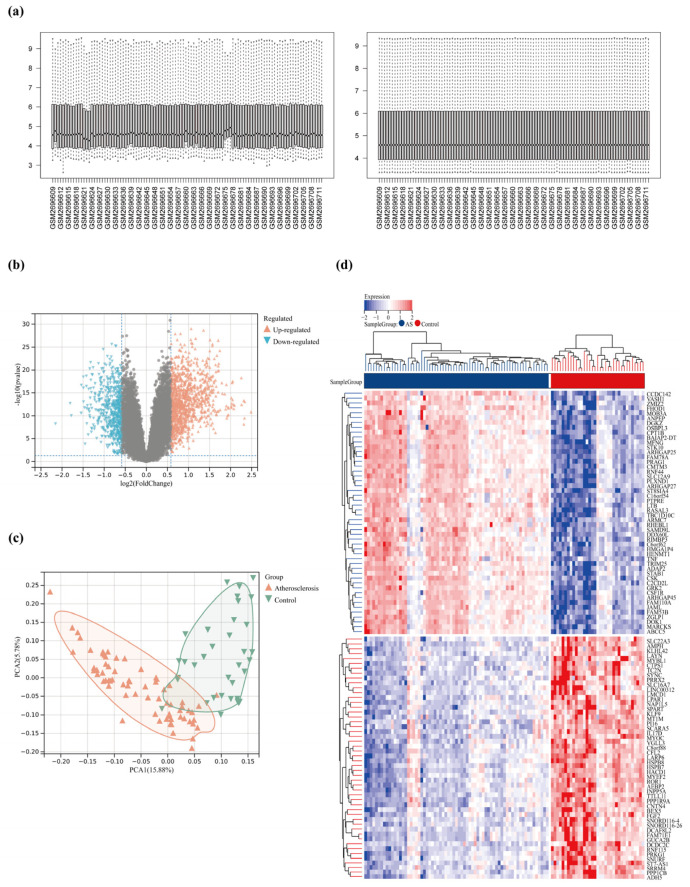
(**a**) Box plots of raw data normalized between samples. (**b**) Volcano plot of DEGs. (**c**) PCA plots; distribution of DEGs in normal and AS groups. (**d**) Heat map of DEGs.

The GEO database was used to acquire the AS clinical samples GSE100927 (GPL17077), including 69 atherosclerotic and 35 normal samples. RNA was abstracted and hybridized to microarrays based on the 104 atherosclerosis patient samples. The DEGs of microRNA data were obtained using R. De-batch effects were applied to all samples (Figure 3a). Under the standards of |log2 (fold change)| > 1.5 and *p* < 0.05, 1630 DEGs were obtained, including 1066 upregulated genes and 564 downregulated genes. Volcano plots and PCA plots were produced to display the DEG assignments (Figure 3b,c). A heatmap showed DEGs expression patterns and relative consistency within groups (Figure 3d).

### 2.5. Gene Functional Enrichment Analysis

**Figure 4:**
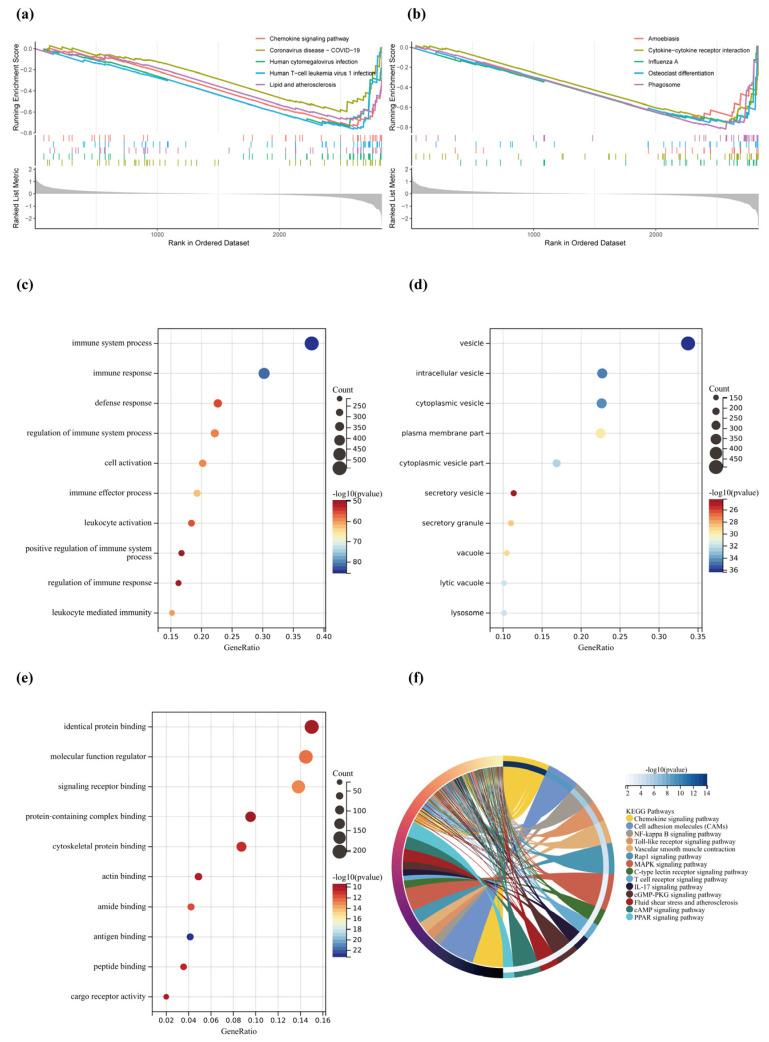
(**a**,**b**) GSEA analysis of DEGs. (**c**) Bubble chart of the biological process terms. (**d**) Bubble chart of the cellular component terms. (**e**) Bubble chart of the molecular function terms. (**f**) KEGG enrichment analysis of DEGs.

GSEA identified the DEGs to be closely associated with chemokine signaling pathways, COVID-19, human cytomegalovirus infection, human T cell leukemia virus 1 infection, lipid and atherosclerosis, cytokine-cytokine receptor infection, and phagosome (Figure 4a,b). GO analysis showed the DEGs in the biological process (BP) terminology to be primarily high in the immune system process, immune response, defense response, and cell activation (Figure 4c). The cellular component (CC) was high in the vesicle, intracellular vesicle, cytoplasmic vesicle, and cytoplasmic vesicle part (Figure 4d). In addition, the terminology “molecular function (MF)” was used for identical protein binding, signaling receptor binding, protein-containing complex binding, and cytoskeletal protein binding (Figure 4e). According to the findings of the KEGG enrichment analysis, DEGs were primarily enriched in the chemokine signaling pathway, cell adhesion molecules (CAMs), NF-kappa B signaling pathway, MAPK signaling pathway, fluid shear and atherosclerosis, cGMP-PKG signaling pathway, and PPAR signaling pathway (Figure 4f, Table 4).

### 2.6. Construction of Co-Expressed Gene Modules

**Figure 5:**
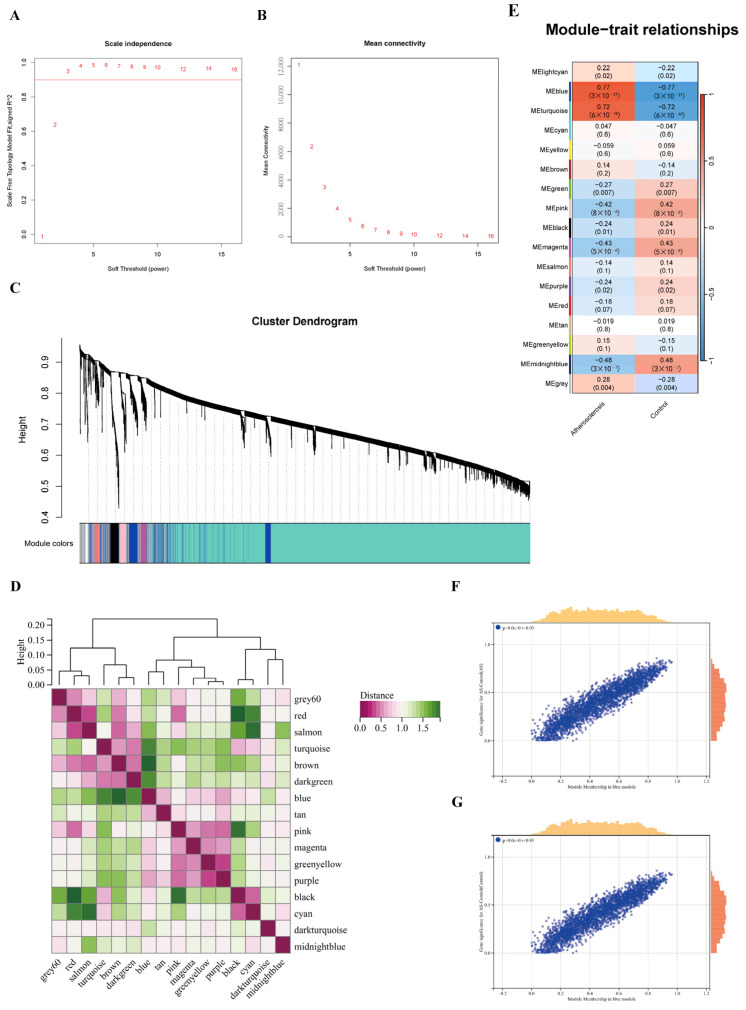
Process of WGCNA. (**A**) Scale-free index analysis for soft-threshold power. (**B**) Mean connectivity analysis for various soft-threshold powers. (**C**) Module cluster dendrogram based on a dissimilarity measure (1-TOM). (**D**) Collinear heat map of module feature genes. Rose red color indicates a high correlation, and the green color indicates opposite results. (**E**) Heat map of module–trait relationships. Red represents positive correlations and blue represents negative correlations. (**F**) MM vs. GS scatter plot of AS. (**G**) MM vs. GS scatter plot of control.

WGCNA was utilized to locate gene modules co-expressed by several genes based on the AS dataset. Samples were initially taken from both datasets and split into two distinct groups: the control group and the atherosclerosis group. No outliers were identified in either group. To guarantee a biologically meaningful scale-free network, 3 was then chosen as the soft threshold power *β* and given a scale independence of >0.8 (Figure 5A–C). In all, 16 relevant co-expression modules were found by using dynamic branch-cutting techniques and hierarchical clustering analysis of gene tree diagrams (Figure 5D). Multiple modules were found to be associated with AS, as can be seen in the module–trait correlation studies. A total of 397 genes were screened in the blue module for upcoming follow-up research after the results were found to indicate that the blue module had the strongest correlation with atherosclerosis (scatter plot) (Figure 5E–G).

### 2.7. Screening and Validation of Hub Genes

**Figure 6:**
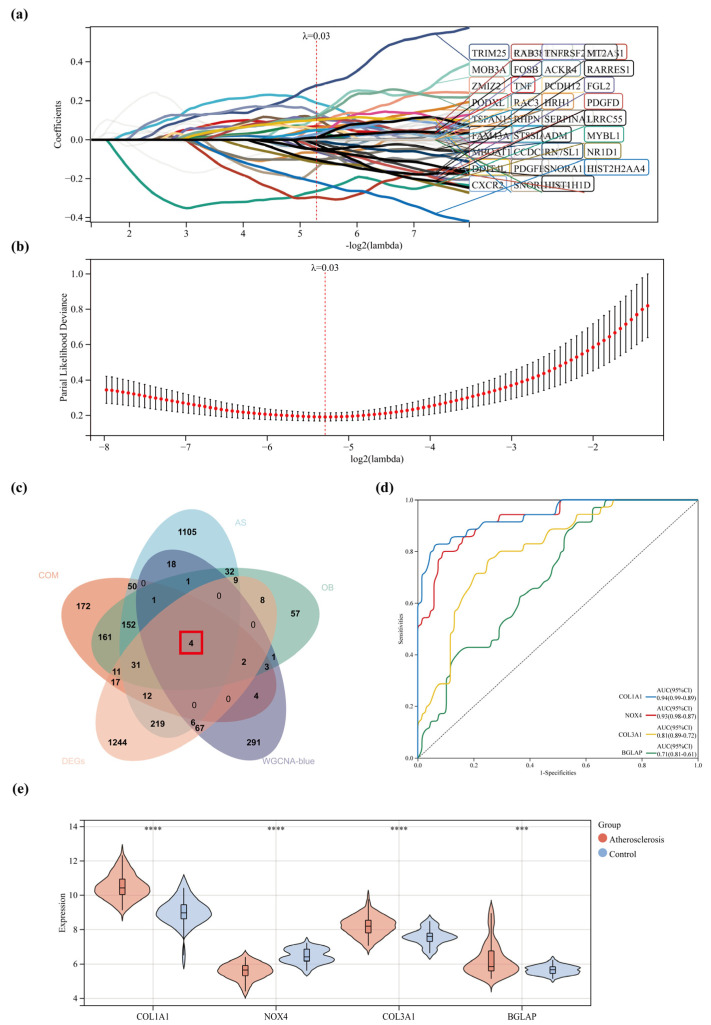
(**a**,**b**) Adjustment of feature selection in the minimum absolute shrinkage and selection operator model (Lasso). Different colors represent different genes. (**c**) Venn diagram obtained four overlapping targets for drug COM and OB with disease AS and DEGs. (**d**) ROC curves of hub genes in the GSE100927 dataset. (**e**) Expression levels of the four hub genes in the GSE100927 dataset. * represents comparing the atherosclerosis group with the control group. *** represents *p* < 0.001, and **** represents *p* < 0.0001.

Four overlapping genes of COM, OB, AS, DEGs, and blue module genes were obtained from the Venn analysis. The four genes were then used as the hub genes: COL1A1, NOX4, COL3A1, and BGLAP (Figure 6c). From the significantly different univariate, 38 predictor genes were selected using LASSO regression analysis, which included COL1A1 among the four hub genes (Figure 6a,b). Patient datasets with and without atherosclerosis were used to confirm the expression values of the four hub genes. It was found that the atherosclerotic plaques of AS patients had much higher COL1A1, COL3A1, and BGLAP expression levels than the healthy controls (*p* < 0.001), while the NOX4 expression level was found to be considerably lower than that of the healthy controls (*p* < 0.001) (Figure 6e). ROC curve analysis was then performed as a means of confirming the clinical relevance of the four hub genes. According to the ROC curve analysis, all four hub genes had an area under the curve (AUC) of less than 0.7, with COL1A1 having the highest AUC value (AUC, 0.94) and BGLAP having the lowest (AUC, 0.71) (Figure 6d).

### 2.8. Immunocyte Infiltration Analysis

**Figure 7:**
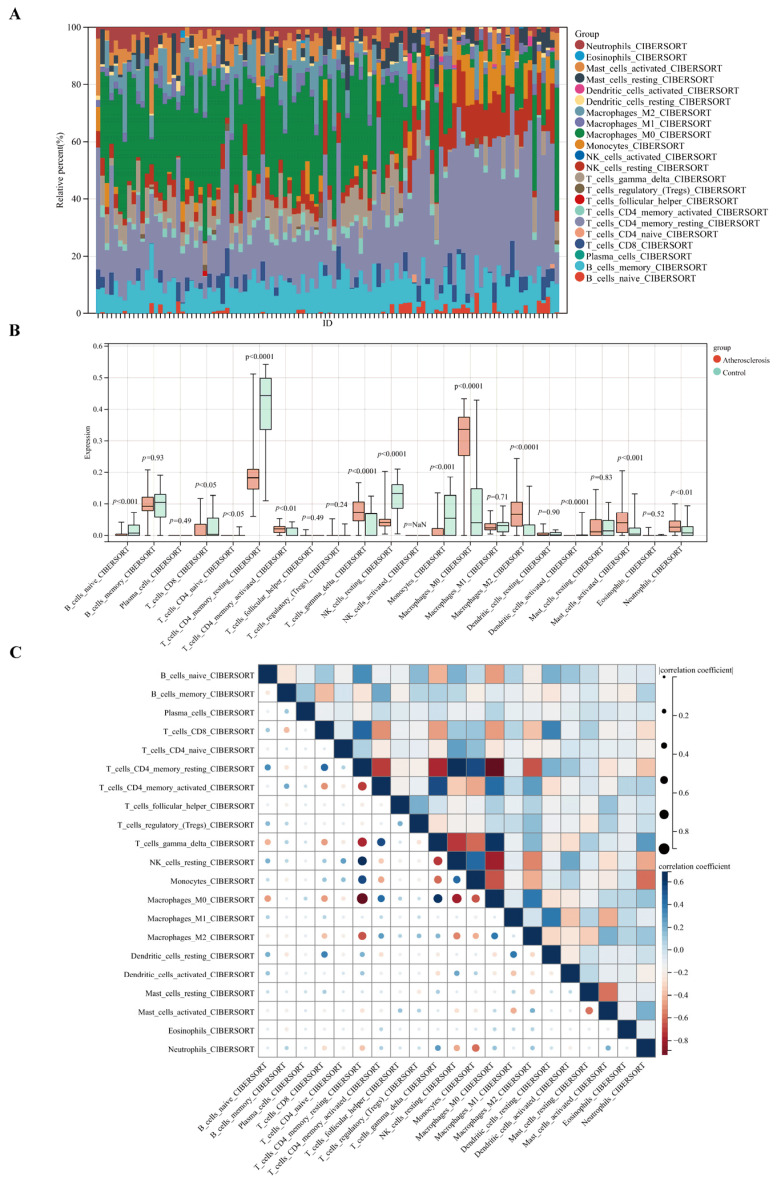
Immune cell infiltration analysis between the AS and control group in the GSE100927 dataset. (**A**) Histogram showing the distribution of 22 immune cell infiltrates. (**B**) Boxplot showing the differences of infiltrated immune cells between subtypes. (**C**) Heat map depicting abundance between different immune cell subtypes.

The abundance of 22 lymphocytes that penetrated the atherosclerotic plaques in both groups was characterized using the CIBERSORT method. Figure 7A,B show the distribution of immunological cell types in all samples from both groups, in addition to the variation in the number of invading lymphocytes in the two groups, which is consistent with the correlation study results. Plaques from AS patients were infiltrated with higher levels of naïve B cells, CD8+ T cells, resting CD4+ memory T cells, activated CD4+ memory T cells, gamma delta T cells, resting NK cells, monocytes, M0 macrophages, M2 macrophages, activated mast cells, and neutrophils in comparison to the healthy controls. The 22 immunological cell types were found to be correlated with each other, as can be seen in Figure 7C, and resting CD4+ memory T cells were found to have the highest negative correlations with M0 macrophages (r = −0.89, *p* < 0.05), while resting CD4+ memory T cells displayed the highest positive correlations with resting NK cells (r = 0.69, *p* < 0.05) (Figure 7C).

### 2.9. Molecular Docking

The high-resolution molecular crystal structures of proteins and ligands were downloaded from the RCSB Protein Database (PDB) [41], AlphaFold Protein Structure Database [42], and PubChem databases. The four hub genes COL1A1 (PDB ID: 5K31), NOX4 (AlphaFold ID: Q9NPH5), COL3A1 (PDB ID: 4AE2), and BGLAP (AlphaFold ID: P02818), and eight active ingredients (quercetin, apigenin, beta-carotene, kaempferol, luteolin, sitosterol, naringenin, and salvigenin) were then taken to perform molecular docking using AutoDock4.2.6 software. The binding energy is shown in Table 5. The binding energy decreased as the stability of the ligand-receptor interaction increased. A more stable docking of all the active components and the four hub genes was discovered based on the binding energy. Following docking, PyMOL 2.5 software was used for visualizing the results into a molecular docking plot, which is shown in the Supplementary Materials. A more stable docking of all the active ingredients of COM and OB top8 and the four hub genes was discovered based on the binding energy.

### 2.10. Molecular Dynamics Simulation

Among the molecular docking results, the best ligands for binding to the hub genes were selected for further MD simulations. In this study, based on the docking results, BGLAP-Naringeni, COL1A1-Apigenin, COL3A1-Sitosterol, and NOX4-Quercetin were subjected to molecular dynamics simulation analyses for 50 ns to evaluate the motion, trajectory, structural features, binding potential, and conformational changes of molecules, which is shown in Table 6.

**Figure 8:**
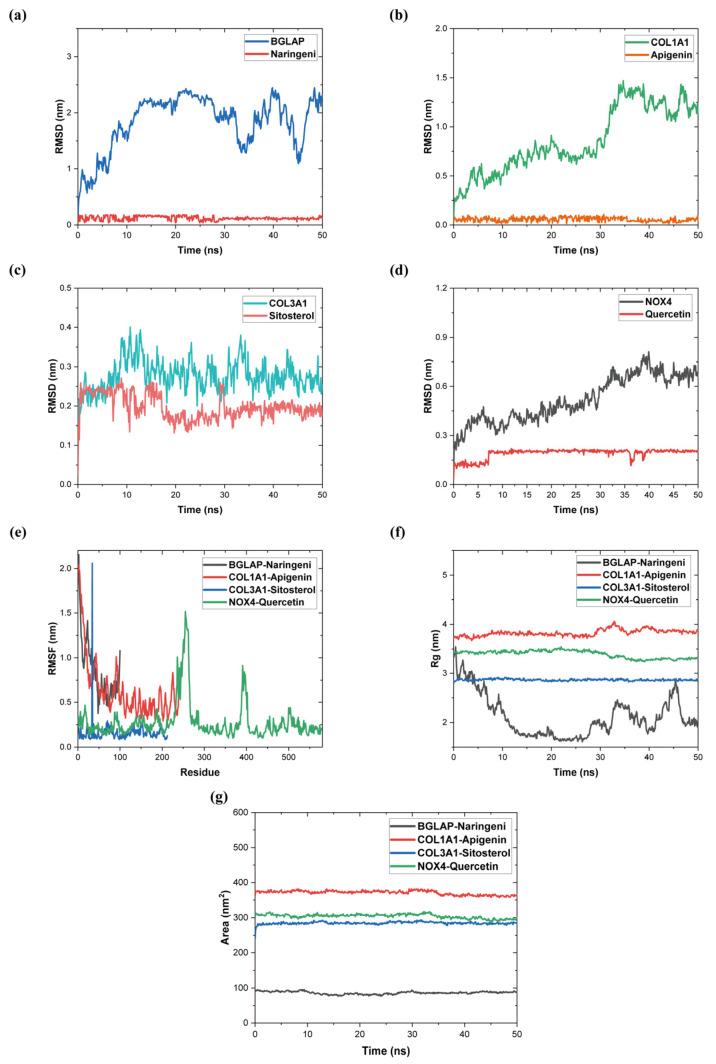
(**a**) RMSD diagram of BGLAP-Naringeni complexes. (**b**) RMSD diagram of COL1A1-Apigenin. (**c**) RMSD diagram of COL3A1-Sitosterol. (**d**) RMSD diagram of NOX4-Quercetin. (**e**) RMSF diagram. (**f**) Rg diagram. (**g**) SASA analysis diagram.

The root mean square deviation (RMSD), as a metric in MD simulations, can be used to measure the stability and flexibility of the proteins and ligands in equilibration and the distances between protein skeletons and atoms. Figure 8a–d illustrates the RMSD trends for four groups of protein–ligand complexes. The results indicated that the average RMSD values of BGLAP-Naringeni, COL1A1-Apigenin, COL3A1-Sitosterol, and NOX4-Quercetin were 1.828, 0.860, 0.276, and 0.520 nm, respectively. This result demonstrated that the ligand was highly stabilized within the macromolecular cavity throughout the simulation time of 50 ns.

The root mean square fluctuation (RMSF) of a protein residue indicates the root-mean-square displacement of the residue in the protein conformations, reflecting the degree of freedom of the protein. Figure 8e displays the RMSFs of the molecular dynamic simulation of the protein–ligand complexes of the four groups. The results showed that the average RMSF values of the four sets of protein–ligand complexes ranged from 0.161 to 0.861 nm. In particular, the BGLAP-Naringeni protein chain was relatively short but fluctuated greatly; COL1A1-Apigenin fluctuated at residues 9–27; COL3A1-Sitosterol fluctuated greatly only at residues 62; and NOX4-Quercetin fluctuated at residues 234–265 and 389–402 and with more stability at the remaining residues. Overall, the COL3A1-Sitosterol binding stability was better, with a smoother overall movement trend, while the remaining three groups of proteins and ligands showed average binding stability.

The radius of gyration (Rg) describes the change in tightness of protein–ligand complexes. It points to the expanding and collapsing of proteins in molecular dynamics simulations. The average Rg values of the four groups of protein–ligand complexes were 2.114, 3.826, 2.868, and 3.392 nm, respectively. The results indicated that except for BGLAP–Naringeni, the protein–ligand complexes were structurally similar and stably bound. In Figure 8f, the Rg values of each of the complexes during the simulation are shown.

Surface area available for solvent (SASA) characterizes the interactions of protein–ligand complexes with solvents and relates them to the surface area of an accessible solvent. In addition, it can predict the structural alterations that have occurred during the entire interaction. As shown in Figure 8g, the average SASA values of the four groups of protein–ligand complexes were 86.146, 370.894, 284.400, and 304.134 nm^2^. The SASA values of the four sets of protein–ligand complexes remained fairly stable over the 50 ns simulation time, indicating that the conformations of the proteins were not changed.

**Figure 9:**
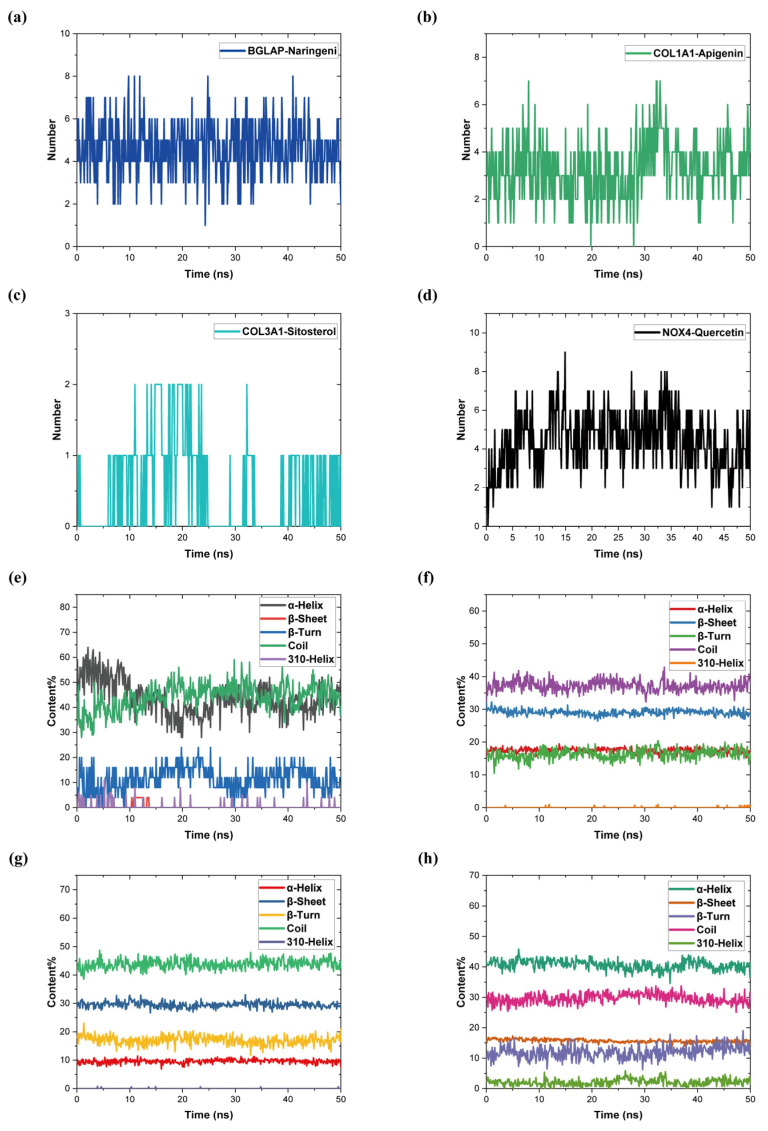
(**a**) Hydrogen bonds diagram of BGLAP-Naringeni. (**b**) Hydrogen bonds diagram of COL1A1-Apigenin. (**c**) Hydrogen bonds diagram of COL3A1-Sitosterol. (**d**) Hydrogen bonds diagram of NOX4-Quercetin. (**e**) SSE analysis diagram of BGLAP-Naringeni. (**f**) SSE analysis diagram of COL1A1-Apigenin. (**g**) SSE analysis diagram of COL3A1-Sitosterol. (**h**) SSE analysis diagram of NOX4-Quercetin.

Hydrogen bonding between proteins and ligands is important for keeping molecules in the active site cavity. For a more detailed characterization of the hydrogen bonding between protein and ligand over all the MD simulations, we calculated the hydrogen bonding for all complexes over a simulation time of 50 ns. The results in Figure 9a–d indicate that the hydrogen bonds for protein and ligand binding were always available during the MD simulations.

Finally, secondary structure element (SSE) analysis was performed on the proteins over the entire trajectory, and the results are shown in Figure 9e–h. In the protein secondary structure, four groups of protein–ligand complexes of α-helix, β-sheet, β-turn, coil, and 310-helix are represented by five different colors. The results indicated that the total proportion of SEEs in the BGLAP-Naringeni complex fluctuated greatly, and its secondary structure and spatial organization were unstable. Except for BGLAP-Naringeni, the total percentage of SSEs in the remaining three groups did not significantly change, and most of the individual SSEs showed stability over all the simulations.

### 2.11. Experimental Validation In Vitro and In Vivo

#### 2.11.1. COM-OB Total Flavonoid Combination Effectively Enhances Cell Viability and Cell Migration and Reduces Lipid Deposition

**Figure 10:**
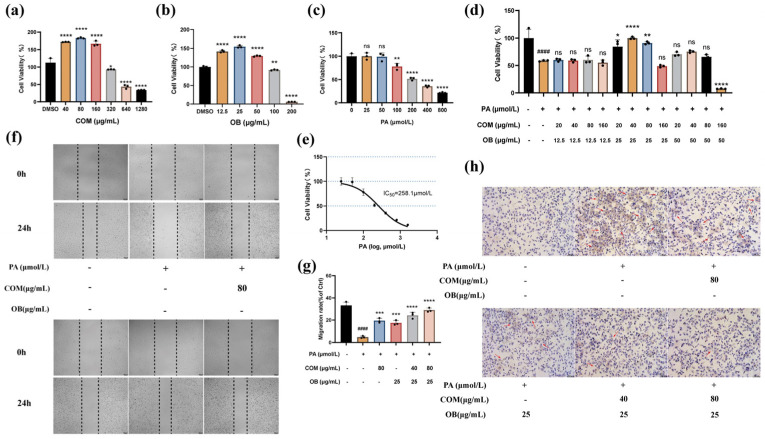
The results of the CCK-8 assay, cell migration assay, and oil red O staining. (**a**–**e**) HUVEC were treated by different concentrations of drugs and then cell viability was assayed by CCK-8 kit. (**f**–**g**) Cells were treated with different concentrations of drugs for 24 h, and cell spacing was measured at 0 h and 24 h with statistical analysis. (**h**) Oil red O staining to analyze lipid deposition in cells treated with different concentrations of drugs. All values are expressed as mean values ± SD. ^#^ represents comparison with the blank group, #### represents *p* < 0.0001, and * represents comparison with the model group. * represents *p* < 0.05, ** represents *p* < 0.01, *** represents *p* < 0.001, and **** represents *p* < 0.0001. ns represents statistically insignificant.

The Cell Counting Kit-8 (CCK8) kit was used to detect the effects of COM and OB on cell viability, PA damage to HUVEC, and the effect of COM-OB total flavonoids combination on PA-induced lipid damage in HUVEC. The results of CCK-8 in Figure 10a–e showed that the COM-OB total flavonoids combination was able to significantly attenuate PA-induced cell damage, which was better than that of the two drugs alone, and the optimal concentration of the combination was determined. Based on the results of the CCK-8 assay, we confirmed by cell migration assay and oil red O staining assay that the COM-OB total flavonoids combination significantly enhanced the migration ability of HUVEC, with a significant increase in cell migration rate, and was able to significantly reduce PA-induced intracellular lipid deposition (Figure 10f–h).

#### 2.11.2. Effect of COM-OB Total Flavonoid Combination on Basic Indexes and Lipid Deposition in Zebrafish

**Figure 11:**
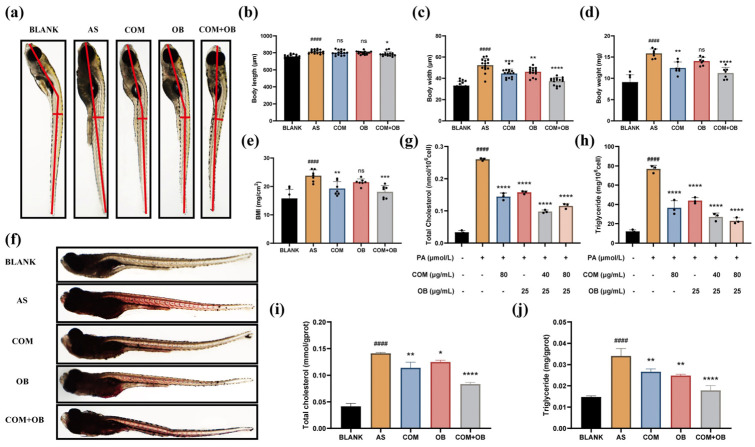
(**a**–**e**) Body length, body width, body weight, and the BMI of *zebrafish* were measured and statistically analyzed after administration of different concentrations of drugs. (**f**) Oil red O staining to analyze lipid deposition in high-cholesterol *zebrafish* treated with different concentrations of drugs. (**g**–**j**) The TC and TG levels were measured and statistically analyzed in PA-induced cells and high-cholesterol *zebrafish*. # represents comparison with the blank group, and * represents comparison with the AS group. #### represents *p* < 0.0001, * represents *p* < 0.05, ** represents *p* < 0.01, *** represents *p* < 0.001, **** represents *p* < 0.0001, and ns represents statistically insignificant.

*Zebrafish* 5 days of fertilization (5 dpf) juveniles were modeled for high cholesterol using a 5% high cholesterol diet fed for 10 days. The basic indexes of *zebrafish* were measured after administration, and the results showed that the combination of total flavonoids by COM-OB could effectively improve the growth of body length and width and reduce the body weight, and the BMI was significantly reduced (Figure 11a–e). Changes in the level of neutral lipid deposition in *zebrafish* were detected using the Oil Red O staining kit. The results in Figure 11f showed that COM-OB total flavonoids conjugation resulted in a significant reduction in the large amount of neutral fat deposits accumulated in the aorta of *zebrafish*.

#### 2.11.3. COM-OB Total Flavonoid Combination Effectively Reduces TC and TG in Cells and Zebrafish

The biochemical kits were used to detect the TG and TC contents in HUVEC and *zebrafish*. The results in Figure 11g–j showed that the COM-OB total flavonoids combination resulted in a significant reduction of TG and TC content in PA-induced cells and high-cholesterol *zebrafish*.

#### 2.11.4. Validation of Hub Genes Protein Expression

**Figure 12:**
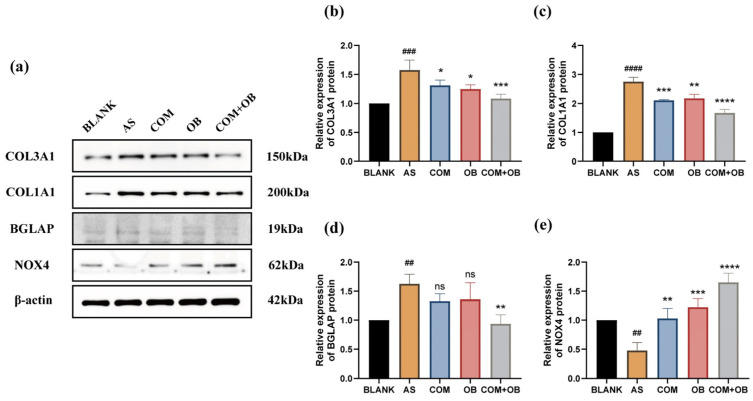
The results of Western blot. (**a**) Effects of different concentrations of drugs on the expression of COL3A1, COL1A1, BGLAP, and NOX4 protein. (**b**–**e**) The statistical analyses of protein expression of the four genes. # represents comparison with the blank group, and * represents comparison with the AS group. ## represents *p* < 0.01, ### represents *p* < 0.001, and #### represents *p* < 0.0001. * represents *p* < 0.05, ** represents *p* < 0.01, *** represents *p* < 0.001, **** represents *p* < 0.0001, and ns represents statistically insignificant.

According to the results of the bioinformatics section, four hub genes, COL1A1, COL3A1, BGLAP, and NOX4, were obtained for COM-OB total flavonoids conjugate treatment of AS, and protein expression of intracellular hub genes was detected by Western blot. The results showed that, consistent with the results of bioinformatic analysis, the COM-OB total flavonoids combination resulted in significant down-regulation of intracellular COL1A1, COL3A1, BGLAP protein expression, and significant up-regulation of NOX4 protein expression in Figure 12a–e. Nevertheless, the exact signaling pathways affected by the regulation of hub gene expression need further intensive study, which will be the focus of our next research.

## 3. Discussion

In all, 50% of all deaths that are caused by cardiovascular disease can be attributed to coronary artery disease (CAD). This outcome is avoidable if the disease is recognized early, and preventive health care plays a vital role in the fight against CAD. CAD is caused by AS of the coronary arteries [43]. AS progression and the rupturing of atherosclerotic plaques cause occlusion of the coronary arteries. Although state-of-the-art technology and the latest secondary prevention therapies are widely available, the burden of recurrence following CAD remains unacceptable. As a consequence, AS remains at the forefront of the challenges to a long and healthy life on a global scale, from both clinical and public health challenge perspectives [44].

The advantages of TCM for AS treatment should not be underestimated due to its rich targets, significant efficacy, and high safety, which have great potential for relieving symptoms and blocking disease progression [15]. *Cydonia oblonga* Mill. (COM) and *Ocimum basilicum* L. (OB) are traditional Uyghur medicines that are regularly used for the treatment of cardiovascular diseases in Xinjiang [23,29]. Our previous study found that the co-administration of their total flavonoids helped prevent AS generation.

In this study, 44 active ingredients and 3302 ingredient targets of two drugs were collected. The ingredient–target network and PPI network were then constructed. According to the PPI network outcomes, AKT1, IL6, and TNF were found to be the top three in terms of degree value. Between atherosclerotic and normal controls, this study identified 1630 DEGs. According to the functional enrichment analysis results, the genes in the GO analysis were mainly enriched in the immune system process, immune response, defense response, vesicle, intracellular vesicle, cytoplasmic vesicle, identical protein binding, molecular function regulator, and signaling receptor binding. KEGG analysis found them to be mainly enriched in the chemokine signaling pathway, cell adhesion molecules (CAMs), NF-kappa B signaling pathway, MAPK signaling pathway, C-type lectin receptor signaling pathway, fluid shear, and atherosclerosis. Based on these DEGs, the results of the WGCNA were combined as a means of identifying hub genes. The WGCNA results showed the blue module to have the most significant association with AS, and 397 genes in the blue module were screened for further study. COL1A1, NOX4, COL3A1, and BGLAP were ultimately chosen as hub genes. The hub genes were verified using LASSO regression analysis and ROC curve analysis. The CIBERSORT algorithm was used to characterize the abundance of 22 immune cells that infiltrated the plaques and normal tissues of atherosclerotic patients. Finally, the molecular docking of eight active ingredients and four hub genes was accomplished using AutoDock software, the results finding all of them to be well binding. Molecular dynamics simulations based on the results of molecular docking showed that the four groups of protein–ligand complexes exhibited a certain stability. The significant therapeutic effect of COM-OB on AS was also confirmed in vitro and in vivo experiments and validated by Western blot experiments on hub genes.

Many plants, vegetables, fruits, cereal grains, and teas contain flavonoids, a class of polyphenolic chemicals with anti-inflammatory, antithrombotic, and/or antioxidant qualities [45]. Flavonoids have attracted a great deal of medicinal interest as potential future therapies due to their potential in AS prevention [46]. Among the flavonols, Quercetin has been investigated the most. Quercetin dramatically decreases the expression of VCAM-1 and ICAM-1 in human umbilical cord endothelial cells (HUVECs) treated with ox-LDL by attenuating the induction of TLR-NF-*κ*B signaling [47]. Kaempferol is also a key flavonol that is linked to a reduced risk of CVD [48]. It was found to be effective in the prevention of lipid oxidation and endothelial dysfunction [49]. According to a recent study, Luteolin suppressed Nox4 and NF-*κ*B, which decreased the formation of ROS in HUVECs [50]. Naringenin exhibits antioxidant properties that are driven by increased hepatic SOD and catalase activity, as well as up-regulation of SOD, glutathione peroxidase (GSH-Px), and catalase transcripts [51]. Elena Serino et al. found Salvigenin to have both lipid-lowering and mitochondria-al-stimulating effects [52]. Numerous studies have found Apigenin to ameliorate diabetes [53], obesity [54], AS [55], and hepatic lipid accumulation in mice [56]. Most of the research supports the suggestion that sufficient Beta-carotene and Vitamin A status is atherosclerosis-protective [57]. Weiping Wu et al. found *β*-Sitosterol to inhibit trimethylamine generation, attenuate AS, reduce inflammatory responses, and enhance antioxidant defenses [58].

PPI networks are feasible tools for understanding cellular functions, disease mechanisms, and drug design. In PPI networks, biological systems are depicted by proteins (i.e., nodes) and their relations (i.e., edges). By constructing PPI networks and analyzing them, we can comprehend the interrelationships of proteins in the cell and thus locate the key factors for regulation. AKT1 is a phosphorylated form-specific substrate of protein kinase B [59] that is a centrally located node in the phosphatidylinositol 3-kinase (PI3K)/AKT signaling pathway and is responsible for the regulation of cell survival [60]. Three known isoforms of AKT that play regulatory roles in protein synthesis, lipid metabolism, and cell proliferation and survival [61]. It has been demonstrated that the secretion of insulin after eating causes PI3K/AKT signaling pathway activation, which can serve to decrease obesity and insulin resistance. When PI3K/AKT is overexpressed or mutated, this can lead to obesity, cancer, and other diseases [62]. An important cytokine in innate immunity, IL (interleukin)-6 performs a variety of physiological actions that are typically connected to host protection and the control, proliferation, and differentiation of immune cells [63,64,65]. Chronic low-grade inflammatory adverse effects are a driver of atherosclerotic onset and progression. Paul M. Ridker et al. proved that IL-6 pathways play a key role in processes that are associated with atherosclerotic plaque onset, progression, and acute rupture [66]. Tumor necrosis factor alpha (TNF*α*, TNF) is a key mammalian immune response mediator and regulator in healthy organizations and diseases [67]. TNF-α was found to function by enhancing the production of hepatic free fatty acid (FFA) [68] and triglycerides (TGs) while reducing the activity of endothelial lipoprotein lipase. This could lead to increased levels of TGs, reduced levels of HDLs, and an increase in the production of highly atherogenic LDL particles. Elevated TNF-*α* levels may induce endothelial dysfunction and later AS [69].

Type I collagen *α*1 (COL1A1) is a member of the collagen family that is involved in epithelial-mesenchymal transition [70]. COL1A1 was demonstrated to be overexpressed in the arteries of animals that are on an atherogenic diet, and this increase is a contributing factor to the inflammation and activation process of the cytokine TGF-*β*, a cytokine underpinning the process of fibrosis through the expression of COL1A1 [71,72]. Anita M., van den Hoek et al. identified COL3A1 as a key hepatic regulator that is associated with lipid metabolism, inflammation, and fibrosis about aortic target genes that are involved in vascular inflammation and atherosclerotic signaling [73]. NOX4 is one of the seven members of the Nox family and has been recognized as a prospective target for cardiovascular disease in recent years [74]. It was demonstrated that endothelial Nox4 protects against atherosclerosis, and the underlying mechanism involves the inhibition of soluble epoxide hydrolase 2 (sEH, gene EPHX2), a pro-inflammatory and atherogenic factor [75]. Osteocalcin (bone gamma-carboxyglutamate protein; BGLAP) is a strongly conservative molecule that is implicated in bone matrix mineralization. It modulates the dynamic aspects of new bone formations and absorption [76]. BGLAP is recognized as one of the most specialized mature osteoblast markers [77]. A growing number of recent studies have observed accelerated AS progression in rheumatoid arthritis (RA) patients [78,79].

This investigation found novel and potential therapeutic medicines and targets for individualized therapy, and in vivo and in vitro testing was performed to validate their therapeutic effects. Following this, the key signaling pathways need to be further explored to understand the molecular mechanisms.

## 4. Materials and Methods

### 4.1. Screening of COM, OB, and AS-Related Targets

The active ingredients of COM and OB were obtained from the literature and active screening. By using the TCMSP platform, the BATMAN-TCM database, the PubChem database, and the Swiss-Target Prediction database, the targets of COM and OB active ingredients were collected and predicted. By using the search term “Atherosclerosis”, the disease targets were filtered by the GeneCards, TTD, and CTD databases.

### 4.2. Network Construction

The active ingredients and targets were entered into Cytoscape 3.8.0 software, and the ingredient–target network was mapped. The ingredient–target network indicates the relationship between the active ingredient of a drug and the disease target of action and is the most central network in network pharmacology analysis. Using the STRING database, interactions between protein and protein were obtained, and the PPI network was then mapped based on Cytoscape 3.8.0. The PPI network exhibits interactions between proteins that provide potential targets for therapeutic intervention in diseases. The protein size was set according to the degree value.

### 4.3. Identification of DEGs

In the GSE100927 dataset, which was obtained from the GEO database, the carotid, femoral, and infrapopliteal arteries were used to extract atherosclerotic lesions and control arteries (from deceased organ donors) without atherosclerotic lesions. After RNA was extracted by them, it was hybridized to microarrays. We de-batched each sample and performed a difference analysis using the R Studio limma package V3.40.6, and the cutoff for identification of DEGs was chosen as |log2 (fold change)| > 1.5 and *p* < 0.05. Volcano plots and heatmaps of the first 50 DEGs in the dataset were produced using the R package ggplot2 V3.3.5 and heatmap V1.0.12. The volcano plots and heatmaps were utilized to be able to clearly demonstrate the distribution of up- and down-regulated differential genes in the samples based on the screening criteria.

### 4.4. Gene Functional Enrichment Analysis

To better visualize the gene expansion of the high-enriched functional pathways, GSEA was performed using the R package. Differences were considered to have statistical significance if the adjusted *p*-value was <0.05. Gene functional enrichment analysis of the data was conducted as a means of validating the possible functions of targets. GO analysis is a prominent method that is used for assigning functions to genes, and it includes three parts: BP, MF, and CC [80]. BP describes the function of the biological target they enable, CC depicts the location of the gene product both within the cell and in its extracellular environment, and MF characterizes the biochemical activity of the gene product. KEGG enrichment analysis is used to analyze the signaling pathways genes are enriched into [81]. The obtained DEGs were imported into the R package Bioconductor ClusterProfiler (Version 3.19), and GO and KEGG enrichment analyses were then conducted.

### 4.5. Construction of Co-Expressed Gene Modules

The WGCNA method helps enable genomic expression studies. Firstly, by using gene expression profiles, the median absolute deviation (MAD) was calculated for individual genes, and the genes in the top 25% with the lowest MAD values were excluded. The outlier genes and samples were removed using the goodSamplesGenes approach in the R package WGCNA (Version 1.71), and the scale-free co-expression network was built. The *β* is a soft threshold parameter that highlights strongly correlated genes and punishes slightly correlated genes. Following the selection of a power of 3, the adjacencies were then transformed into a topological overlap matrix (TOM) and labeled with colors and modular features (MEs). Hierarchical clustering and dynamic tree-cutting feature detection were used by the modules. In addition, correlation coefficients were determined to assess the association of the ME with clinical features [82].

### 4.6. Screening and Validation of Hub Genes

Venn analysis was carried out on the COM and OB targets, AS targets, DEGs, and significant module genes that were obtained from the above network pharmacology and bioinformatics analyses. The resulting overlapping genes were ultimately used as hub genes. The R package glmnet (Version 4.1-7) [83] was used to integrate survival time, status, and genetic expression, and regression analyses were performed using LASSO-cox analysis. The smallest lambda was deemed to be the optimum value. After the hub genes were obtained, the algorithm was assessed by ROC analysis using the R package pROC (Version 1.18.0), and the AUC was calculated to measure the predictive power of the algorithm.

### 4.7. Assessment and Correlation Analysis of Immunological Infiltration

Cell type characterization was performed by imputing a relative subset of RNA transcripts (CIBERSORT) to demonstrate the differences in immunological infiltration between AS plaques and control tissues. To better understand the mechanisms of molecular immunity, correlational analyses were performed as a means of clarifying the relationships between the various immune cells.

### 4.8. Molecular Docking

1. Four hub genes were obtained based on the screening results from earlier network pharmacology and bioinformatics analyses. The 3D structures of the selected proteins were acquired from the PDB and AlphaFold Protein Structure Database, and the PDB format was downloaded. In the 14th Community Wide Experiment on the Critical Assessment of Techniques for Protein Structure Prediction (CASP14), AlphaFold was ranked as the top protein structure prediction method with high prediction accuracy. Although it still has some limitations, the CASP results indicated that AlphaFold has immediate potential to improve our understanding of protein structure and advance biological research. Proteins were then loaded into AutoDock 4.2.6 to remove water and other ligand molecules, add hydrogen, compute Gasteiger partial charges, and assign AD4 type. The structures were then saved in PDBQT format.
2. Based on the above screening results and the comparison of the degree values of the active ingredients, the eight most effective COM and OB ingredients were identified. The structure of the active ingredients was obtained in SDF format from the PubChem database and was converted to mol2 format using OpenBabel software (Version 3.2.2). The ligand was then subjected to modifications in AutoDock4.2.6, and the ligand profile was preserved in PDBQT format.
3. In order to perform molecular docking, the two aforementioned structure files were exported to AutoDock 4.2.6 software. After calculating the minimal binding energy, OpenBabel software was used to convert it to PDB format.
4. Lastly, visualization of the molecular docking plots was performed with PyMOL2.5 software.

### 4.9. Molecular Dynamics Simulation

MD simulations are utilized to examine the binding stability of small molecules to target proteins. MD simulations using GROMACS 2020 software verified the stability of hub genes binding to the ligand [84]. To ensure that the complexes are stabilized in the same system as in the human body (force-fields: amberff14sb-ildn; Integrator: md; leap-frog integration methods; equilibration ensemble: 100 ps NPT and 100 ps NVT; temperature: 300 K, reference temperature, one for each group; pressure: 1.0 bar, reference pressure, one for each group), hub genes (BGLAP, COL1A1, COL3A1, and NOX4) and the ligands (quercetin, apigenin, naringeni, and sitosterol) were simulated on a GPU system for 50 ns by quantifying the RMSD, RMSF, Rg, hydrogen bond, SASA, and SSEs of the hub genes and ligands in the active site of the ligand.

The RMSD is an important parameter for measuring the change in protein structure; it indicates the average deviation between the protein structure and the reference structure during simulation. As a practical matter, the stability and dynamic changes in protein structure can be monitored by calculating the RMSD, which can provide an in-depth understanding of the function and properties of proteins. The ligand fluctuations within the four active protein sites were determined by the RMSD of the MD trajectories [85]. RMSF is a measure of the deviation of atoms from their average position in the structure of a protein or ligand. It is valued for assessing the dynamics and flexibilities of a protein as well as of a ligand molecule. The importance of the RMSF for proteins is that it can provide information about the comparative flexibility of different fractions, and it can be used for predicting protein dynamics and assessing stability. The Rg describes the change in the tightness of protein–ligand complexes [86]. The size of the Rg value is directly related to the stability of the structure. A larger Rg indicates less stability, and a lower Rg indicates higher stability. Structural stability indicates that binding to the ligand does not affect the conformation of the macromolecular protein, and also indicates that the drug ligand binds relatively well to the protein as a complex.

The SASA represents the area of the complex surface in contact with the solvent. In practice, by calculating the SASA, the degree of exposure of the protein molecule to the solvent can be assessed, as well as the solvent accessible to different regions or residues. The lower the SASA value of the complex, the higher the stability. Hydrogen bonding between proteins and ligands is important for keeping molecules in the active site cavity. The SSE describes the composition and distribution of secondary structures, such as *α*-helices and *β*-folds, in complexes of proteins and ligands. By recognizing and analyzing the SSEs, it is possible to gain a deeper understanding of the structural features and spatial organizations of proteins, as well as the relationship between these features and protein functions [87]. It is important for protein structure prediction, structure comparison, and drug design. These complementary analyses further clarified the dynamic behaviors and stability of the protein–ligand complexes under study.

The RMSD and RMSF of protein–ligand complexes were calculated using the gmx rms and gmx RMSF applications. The SASA, hydrogen bonding, and Rg were calculated using the gmx SASA, gmx hbond, and gmx gyrate tools.

### 4.10. Experimental Validation In Vitro and In Vivo

#### 4.10.1. Cell Culture and Cell Viability Detection

HUVEC (human umbilical vein endothelial cells) (American Type Culture Collection (ATCC), Manassas, VA, USA) were cultured in a DMEM medium (Thermo Fisher Scientific, Waltham, MA, USA) with 10% FBS, 100 mg/mL streptomycin, and 100 U/mL penicillin (Solarbio, Beijing, China) at 37 °C in 5% CO_2_. Total quince flavonoids and total basil flavonoids were obtained from phytomedicinal herbs, respectively. Palmitic acid solution (PA) was prepared and administered to the HUVEC at different concentrations for modeling. The Cell Counting Kit-8 (CCK-8, bs-0764P, Bioss, Beijing, China) method was used to assay the cell viability according to the instructions.

#### 4.10.2. *Zebrafish* Culture and Basic Indicator Testing

Three-month-old AB-wild-type adult *zebrafish* were purchased from the Institute of Aquatic Biology, Chinese Academy of Sciences (Wuhan, China). Males and females were placed in a 1:1 ratio in a clear glass aquarium and separated by a baffle, and the following day the baffle was removed in the early morning to mix the spawn. The juveniles were collected from 5 days of incubation at 28 °C, and those with normal development were randomly grouped by selecting them under a body microscope. A 5% high-cholesterol diet (Xiaoshuyoutai Biotechnology Co., Ltd., Beijing, China) and corresponding concentrations of total flavonoids were administered. Ten days after administration, basic measurements of *zebrafish* in each group were taken.

#### 4.10.3. Cell Migration and Oil Red O Staining

HUVEC were inoculated in 6-well plates at a density of 3 × 10^5^ cells/well and cultured overnight. The original medium was discarded, and the cell layer was scored along the scribe line with a 200 μL lance tip perpendicular to the cell plane. After administration, the cells were incubated in an incubator for 0 h, 24 h, and 48 h, washed three times with PBS, observed and photographed under a microscope, and the area of the scratches was calculated by Image J. The cells were stained by adding Oil Red O fixative, Oil Red O staining solution, and Mayer hematoxylin staining solution, respectively, according to the instruction of the kit (Solarbio, Beijing, China), and finally, the buffer was added and photographed under the microscope for observation.

#### 4.10.4. Biochemical Indicator Testing

According to the total cholesterol (TC) and triglyceride (TG) content assay kit (Solarbio, Beijing, China) operation guide, the effect of COM-OB total flavonoids on the TC and TG content in PA-induced HUVEC and 5% high cholesterol diet-induced *zebrafish* was examined using biochemical kits.

#### 4.10.5. Western Blotting

HUVEC were inoculated in 6-well plates at a density of 3 × 10^5^ cells/well and cultured overnight. Cells were modeled and treated with drug administration for 24 h. After obtaining the cells, they were lysed in RIPA buffer (Solarbio, Beijing, China), and proteins were extracted. Proteins were loaded onto SDS-PAGE gels for electrophoresis and transferred to PVDF membranes (PVDF; Millipore, Boston, MA, USA). Incubation of primary antibodies (anti-COL3A1, at 1:1000, Abcam, Cambridge, UK; anti-COL1A1, at 1:1000, Cell Signaling Technology, Boston, MA, USA; anti-BGLAP, at 1:1000, Abcam, Cambridge, UK; anti-NOX4, at 1:1000, Proteintech, Wuhan, China) was performed to validate the four hub genes. Proteins were visualized using a chemiluminescent imaging system (Biorad, Hercules, CA, USA).

### 4.11. Statistical Analysis

The experimental results were analyzed statistically using GraphPad Prism 10.0.0 software; data were expressed as mean values ± SD; a *t*-test was used for the statistical analysis of two independent samples; and a one-way ANOVA was used for the statistical analysis of three or more samples. *p* < 0.05 indicates statistical significance.

## 5. Conclusions

In conclusion, this paper provided and validated four hub genes as therapeutic targets for AS treatment with COM-OB using network pharmacology and bioinformatics analysis. Meanwhile, we also verified the significant therapeutic effect of COM-OB co-administration on AS in the results of in vivo and in vitro experiments. It is therefore believed that these eight active ingredients in the Uyghur medicines COM and OB and the four hub genes can be new therapeutic agents and potential targets for AS prevention, diagnosis, and treatment in the future. In the next study, we will focus on the signaling pathways involved in hub genes to further investigate the key molecular mechanisms of the COM-OB combination for AS. This will serve to promote the progress of Chinese medicine in AS treatment.

## 6. Patents

Umar, A.; Zhou, W.; Abdurahman, A.; Abulizi, A.; Naman, A.; Duolikong, A.; Dilixiati, A.; Keremu, A.; Sawuti, A.; Simayi, J. A quince - basil total flavone composition and its application in atherosclerosis. CN110327392B, 2019-07-25.

## Figures and Tables

**Table 1:** The active ingredient targets of COM and OB.

No.	Ingredient	TCMSP	BATMAN	Swiss-Target Prediction	Total
1	Isoquercitrin	5	0	50	55
2	Quercetin 3-galactoside	9	0	22	31
3	Sitosterol	3	96	44	143
4	Rutin	42	4	34	80
5	Amygdalin	6	0	42	48
6	Beta-carotene	19	151	0	170
7	Chlorogenic acid	0	0	6	6
8	Quercetin	299	178	200	677
9	Kaempferol	121	10	200	331
10	Catechin	20	30	0	50
11	Quercetin-3-O-β-d-glucoside	0	0	22	22
12	Kaempferol-3-O-β-d-glucoside	0	0	21	21
13	Nevadensin	17	0	12	29
14	Quercetin-3-O-β-d-galactopyranoside	0	0	22	22
15	Quercetin-3-O-α-l-rhamnoglucoside	0	0	21	21
16	Myricitrin	2	0	22	24
17	Syringin	18	0	29	47
18	Apigenin	77	3	100	180
19	Naringenin	11	27	97	135
20	Genkwanin	13	0	59	72
21	Luteolin	55	0	100	155
22	Eriodictyol	9	7	18	34
23	Epicatechin	6	15	0	31
24	Velutin	0	0	100	100
25	Salvigenin	17	0	100	117
26	Cirsiliol	9	0	100	109
27	Pilosin	0	0	100	100
28	Eupalitin	15	0	100	115
29	Xanthomicrol	17	0	13	30
30	GardeninB	0	0	100	100
31	Hymenoxin	0	0	100	100
32	Molludistin	0	0	4	4
33	Cosmosiin	1	0	22	23
34	Quercetrin	0	0	21	21
35	Luteolin-7-O-glucoside	3	0	22	25
36	Orientin	5	0	4	9
37	Luteolin-7-O-glucuronide	0	0	21	21
38	Quercetin-3-O-galactoside	9	0	22	31
39	Quercetin-3-O-glucuronide	2	0	21	23

**Table 2:** The degree value of active ingredients.

No.	Ingredient	Degree	Betweenness
1	Quercetin	310	0.238451988
2	Apigenin	171	0.027592652
3	Beta-carotene	170	0.09686698
4	Kaempferol	155	0.06398417
5	Luteolin	143	0.015631378
6	Sitosterol	136	0.077195616
7	Naringenin	131	0.039760769
8	Salvigenin	113	0.008192679

Note: Degree value: the number of edges directly connected to that node; Betweenness value: a measure of the intermediary role of the node in the shortest path of the network, nodes with high betweenness centrality play an important role in the communication between different nodes.

**Table 3:** The degree value of the top 10 proteins of PPI.

No.	Protein	Degree	Betweenness
1	AKT1	140	0.0386639853635396
2	IL6	139	0.0370961728791916
3	TNF	138	0.0348905245840174
4	INS	128	0.0312717654742738
5	TP53	126	0.0298558219169182
6	IL1B	125	0.0263218283412141
7	CASP3	121	0.0149378769869302
8	BCL2	118	0.0170485147677625
9	PTGS2	117	0.0291340122024729
10	ESR1	116	0.030196923984057

**Table 4:** The information of top 15 significant KEGG enrichment analysis.

Pathway ID	Pathway	Count	*p* Value
hsa04062	Chemokine signaling pathway	54	7.83181195018207 × 10^−15^
hsa04514	Cell adhesion molecules (CAMs)	38	2.02647264320902 × 10^−9^
hsa04064	NF-kappa B signaling pathway	26	9.83660273222764 × 10^−7^
hsa04620	Toll-like receptor signaling pathway	25	5.01052403629501 × 10^−6^
hsa04270	Vascular smooth muscle contraction	27	0.0000496199445295329
hsa04015	Rap1 signaling pathway	36	0.000154841951809588
hsa04010	MAPK signaling pathway	42	0.00245564583703391
hsa04625	C-type lectin receptor signaling pathway	19	0.0025703743733173
hsa04660	T cell receptor signaling pathway	19	0.0025703743733173
hsa04657	IL-17 signaling pathway	17	0.00420292315856717
hsa04022	cGMP-PKG signaling pathway	26	0.00487633918446109
hsa05418	Fluid shear stress and atherosclerosis	22	0.00756887958413684
hsa04024	cAMP signaling pathway	31	0.00766376826866491
hsa05110	Vibrio cholerae infection	10	0.0137763850285278

**Table 5:** Docking scores of hub genes with ingredients (kcal·mol^−1^).

	CID	BGLAP	COL1A1	COL3A1	NOX4
Quercetin	5280343	−1.45	−1.88	−2.05	−3.19
Apigenin	5280443	−3.02	−4.91	−3.83	−2.82
Beta-carotene	5280489	−4.92	−4.92	−4.45	−4.64
Kaempferol	5280489	−3.34	−2.95	−2.65	−2.55
Luteolin	5280863	−2.73	−2.83	−4.28	−2.27
Sitosterol	5280445	−4.03	−4.07	−5.03	−4.31
Naringenin	222284	−3.35	−4.65	−3.76	−3.23
Salvigenin	439246	−3.31	−3.26	−3.02	−2.45

**Table 6:** Stability analysis of hub genes and ligands by molecular dynamics simulations.

	RMSD (nm)	RMSF (nm)	Rg (nm)	SASA (nm^2^)
BGLAP-Naringeni	1.828	0.861	2.114	86.146
COL1A1-Apigenin	0.860	0.634	3.826	370.894
COL3A1-Sitosterol	0.276	0.161	2.868	284.400
NOX4-Quercetin	0.520	0.280	3.392	304.134

## Data Availability

The datasets (GSE100927, GPL17077) for this study can be found in the Gene Expression Omnibus (GEO) database [https://www.ncbi.nlm.nih.gov/geo/ (accessed on 25 December 2023)]. All the data in this paper support the results of this study, other datasets used and/or analyzed during the current study are available from the corresponding author on reasonable request.

## Supplementary Materials

The following supporting information can be downloaded at: https://www.mdpi.com/article/10.3390/ph17111433/s1, Figure S1: Figure of molecular docking; Table S1: AS Target; Table S2: COM-Target; Table S3: OB-Target; Table S4: Network; Table S5: Type; Table S6: Compound-Degree; Table S7: COM, OB-Degree; Table S8: PPI-Degree; Table S9: COM-OB-AS188 target; Table S10: GSE100927DEGs; Table S11: BGLAP-MD_results; Table S12: COL1A1-MD_results; Table S13: COL3A1-MD_results; Table S14: NOX4-MD_results.
