# Supplementary figures and images for: Searching for Hub Genes of Quince–Basil Co-Administration Against Atherosclerosis Using Bioinformatics Analysis and Experimental Validation
Source: Pharmaceuticals (Basel). 2024 Oct 25;17(11):1433. doi: 10.3390/ph17111433 (PMC11597616; doi:10.3390/ph17111433)

## BGLAP-Apigenin

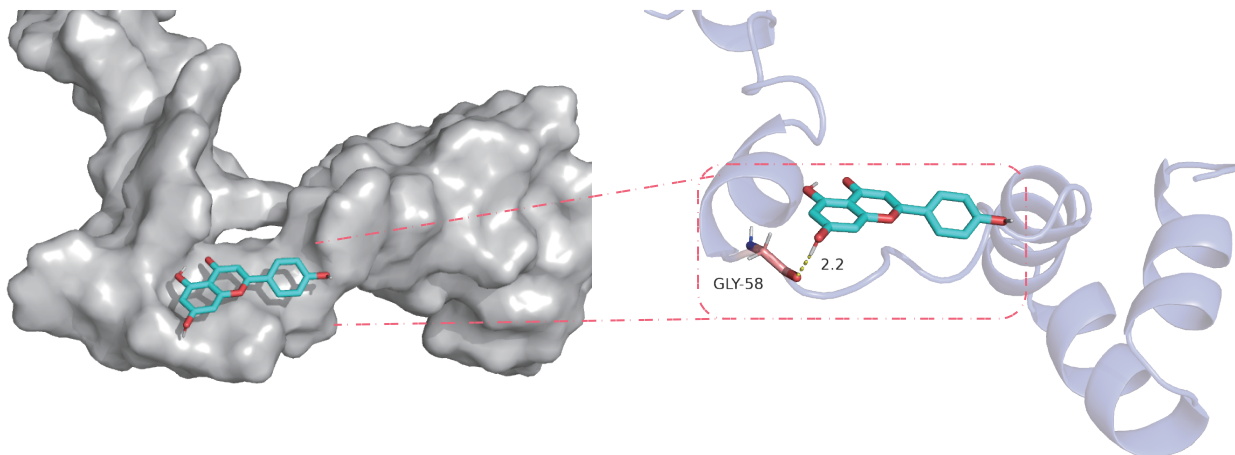

## COL1A1-Apigenin

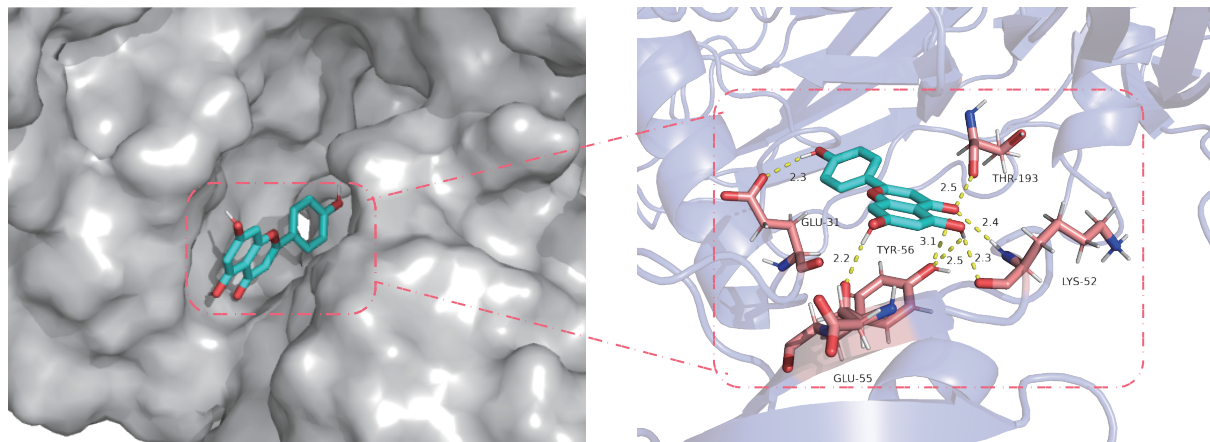

Supplement: Supplementary file 1 [file pharmaceuticals-17-01433-s001.zip › pharmaceuticals-3261423-supplementary/Supplementary File-Figure of molecular docking/BGLAP-Apigenin+COL1A1-Apigenin.pdf]

## BGLAP-Kaempferol

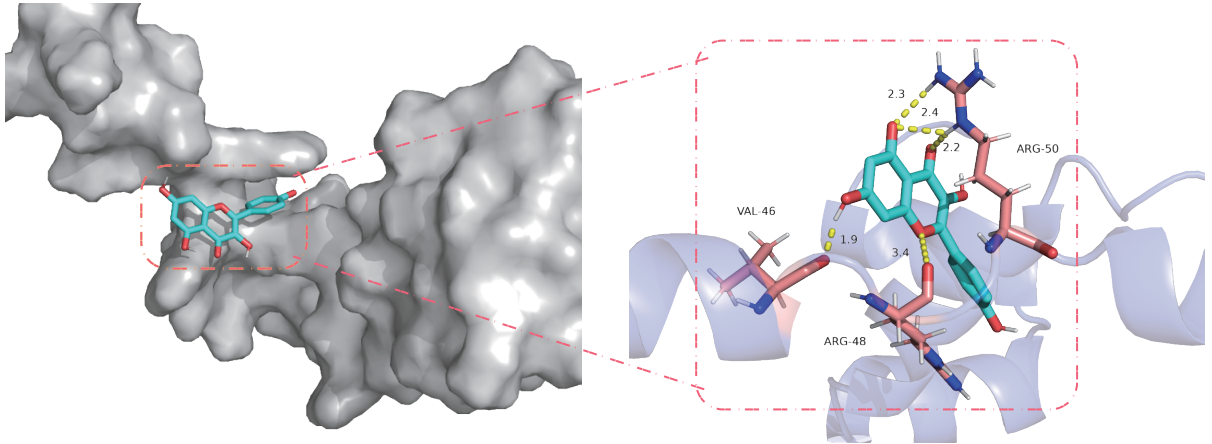

## COL1A1-Kaempferol

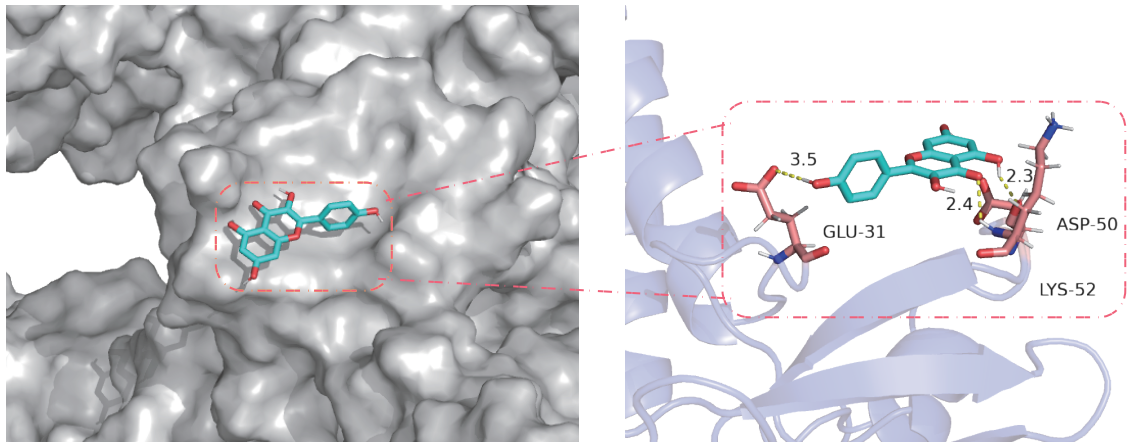

Supplement: Supplementary file 1 [file pharmaceuticals-17-01433-s001.zip › pharmaceuticals-3261423-supplementary/Supplementary File-Figure of molecular docking/BGLAP-Kaempferol+COL1A1-Kaempferol.pdf]

## BGLAP-Luteolin

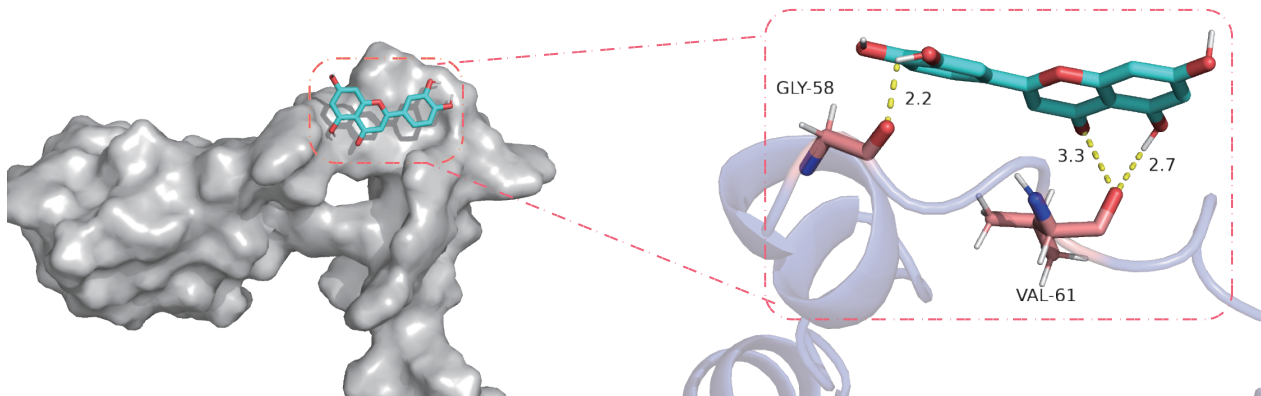

## COL1A1-Luteolin

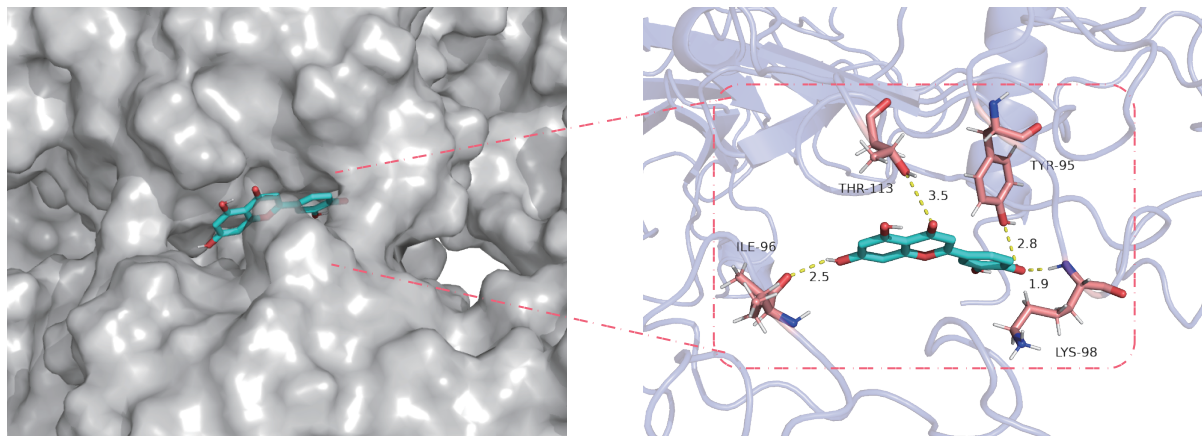

Supplement: Supplementary file 1 [file pharmaceuticals-17-01433-s001.zip › pharmaceuticals-3261423-supplementary/Supplementary File-Figure of molecular docking/BGLAP-Luteolin+COL1A1-Luteolin.pdf]

## BGLAP-Naringenin

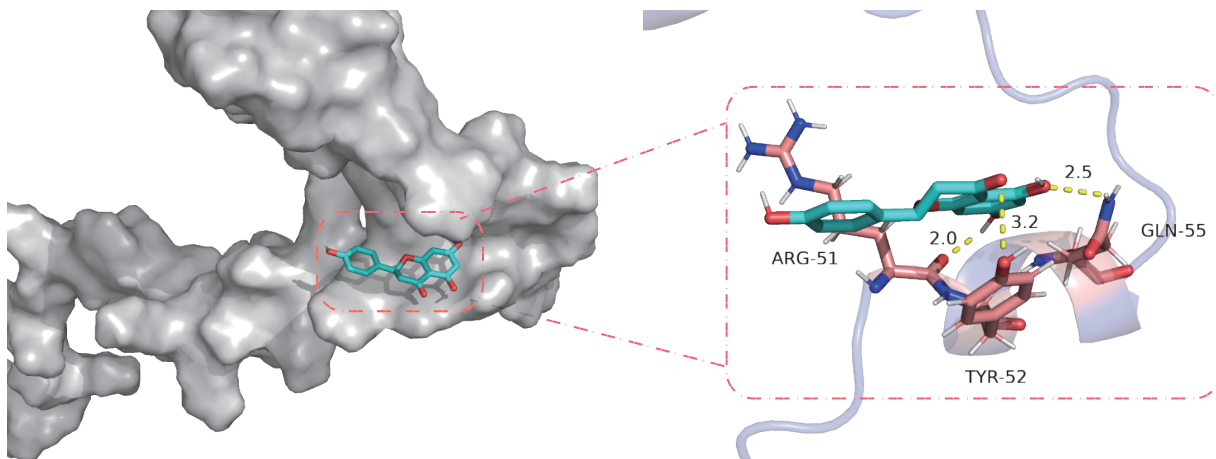

## COL1A1-Naringenin

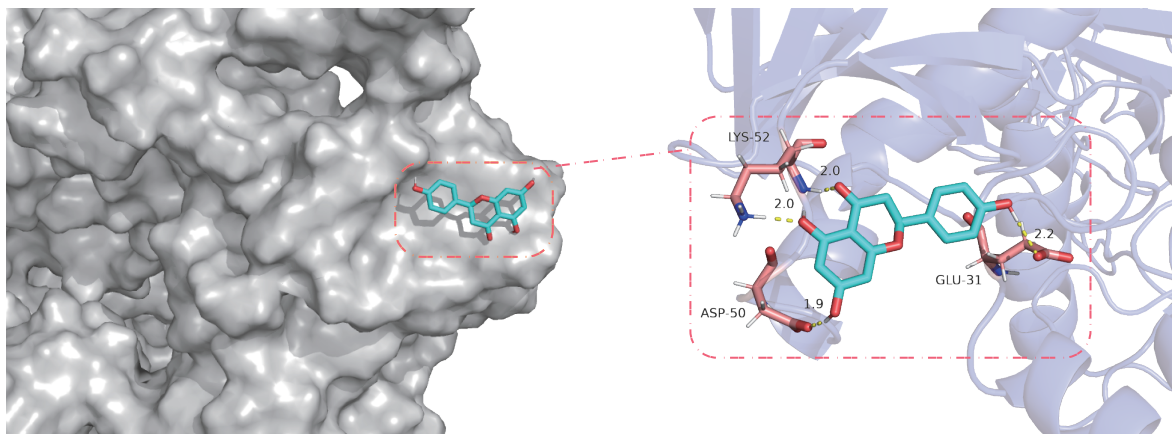

Supplement: Supplementary file 1 [file pharmaceuticals-17-01433-s001.zip › pharmaceuticals-3261423-supplementary/Supplementary File-Figure of molecular docking/BGLAP-Naringenin+COL1A1-Naringenin.pdf]

## BGLAP-Quercetin

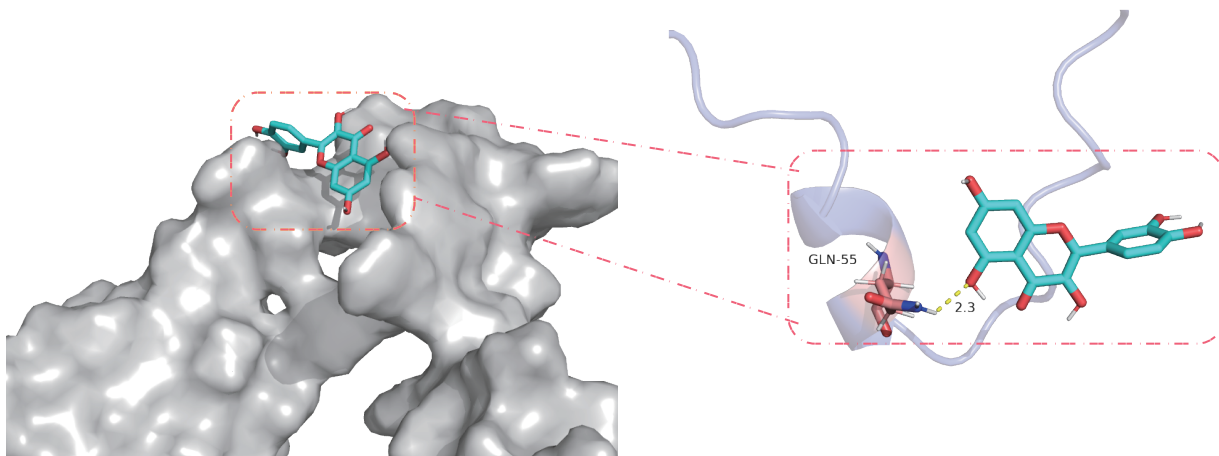

## COL1A1-Quercetin

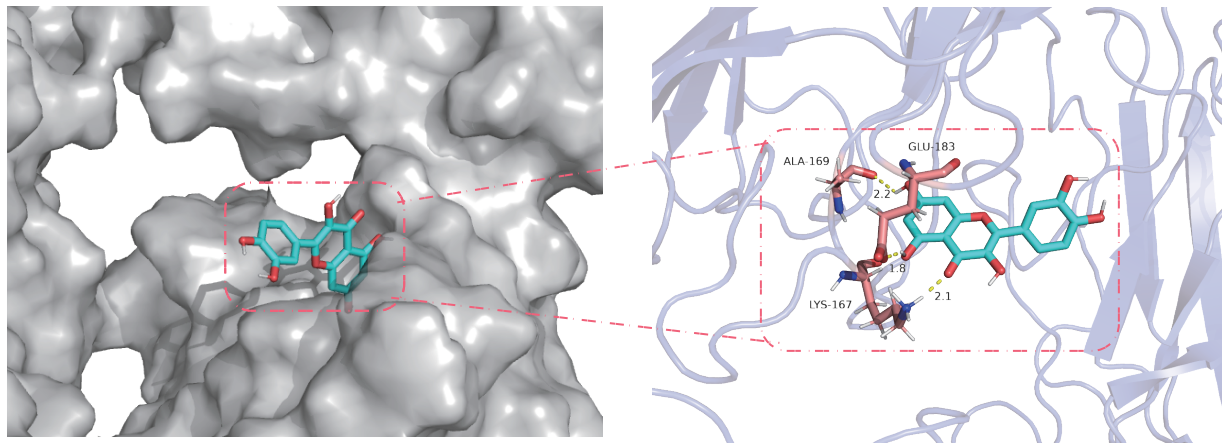

Supplement: Supplementary file 1 [file pharmaceuticals-17-01433-s001.zip › pharmaceuticals-3261423-supplementary/Supplementary File-Figure of molecular docking/BGLAP-Quercetin+COL1A1-Quercetin.pdf]

## COL1A1-Salvigenin

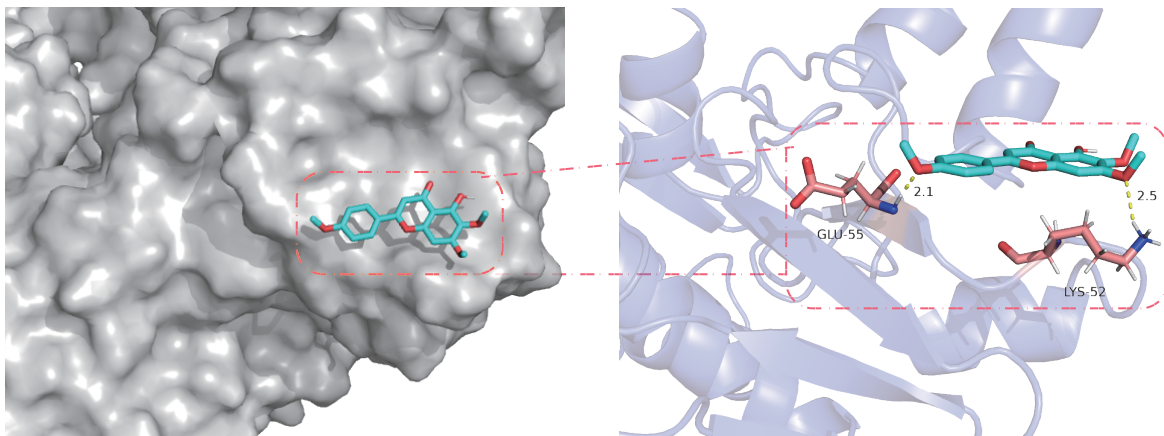

## COL3A1-Salvigenin

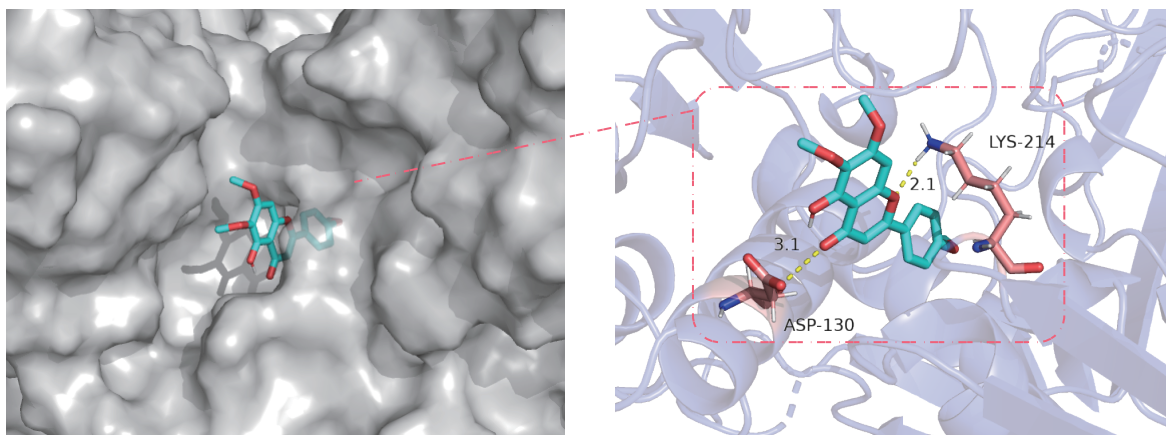

Supplement: Supplementary file 1 [file pharmaceuticals-17-01433-s001.zip › pharmaceuticals-3261423-supplementary/Supplementary File-Figure of molecular docking/COL1A1-Salvigenin+COL3A1-Salvigenin.pdf]

## COL1A1-Sitosterol

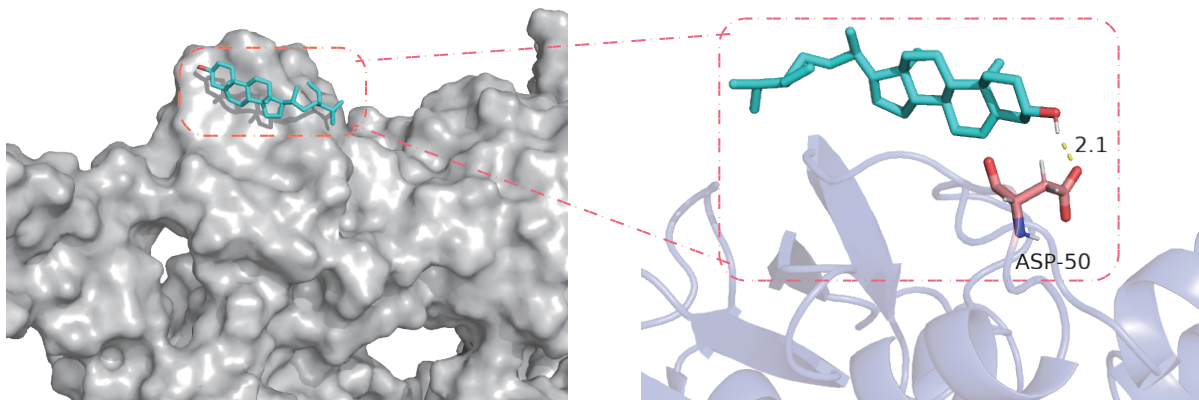

## COL3A1-Sitosterol

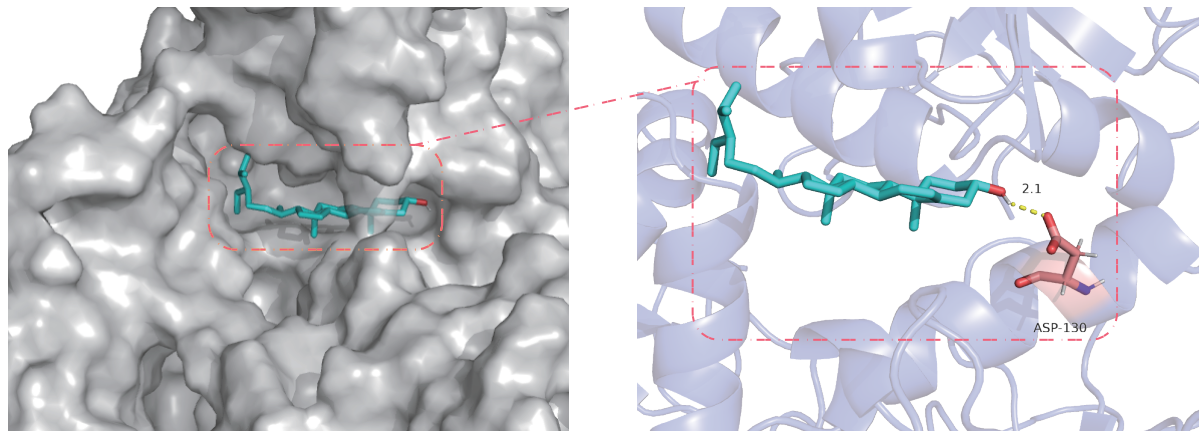

Supplement: Supplementary file 1 [file pharmaceuticals-17-01433-s001.zip › pharmaceuticals-3261423-supplementary/Supplementary File-Figure of molecular docking/COL1A1-Sitosterol+COL3A1-Sitosterol.pdf]

## COL3A1-Apigenin

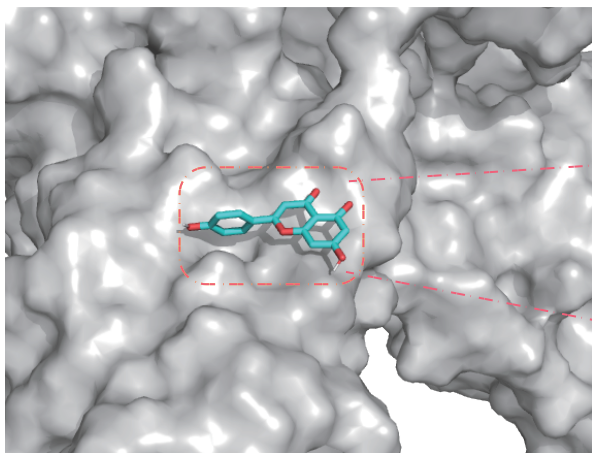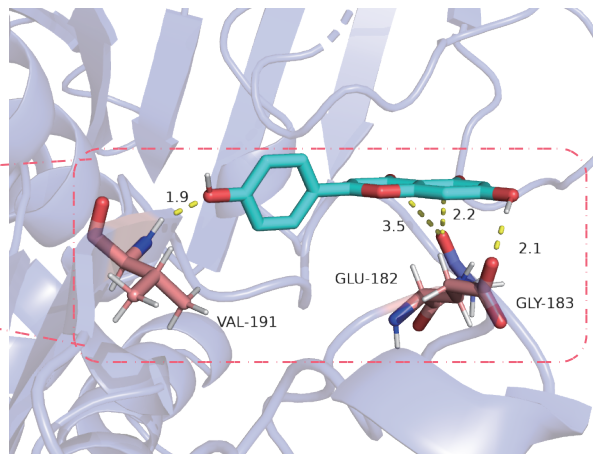

## NOX4-Apigenin

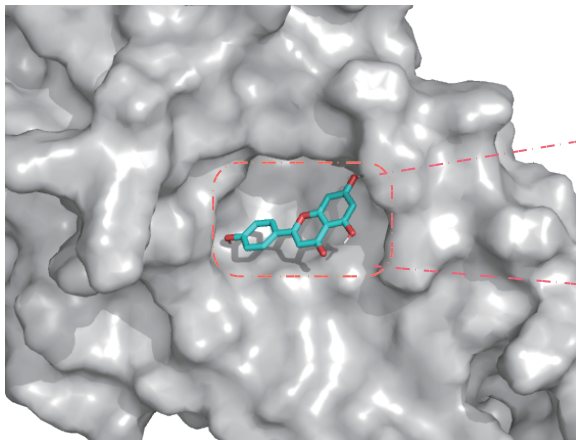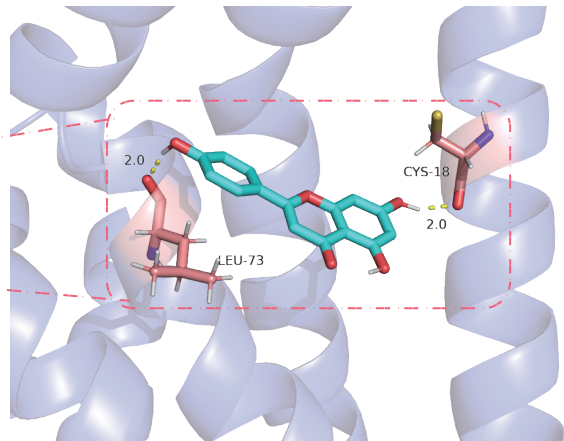

Supplement: Supplementary file 1 [file pharmaceuticals-17-01433-s001.zip › pharmaceuticals-3261423-supplementary/Supplementary File-Figure of molecular docking/COL3A1-Apigenin+NOX4-Apigenin.pdf]

## COL3A1-Kaempferol

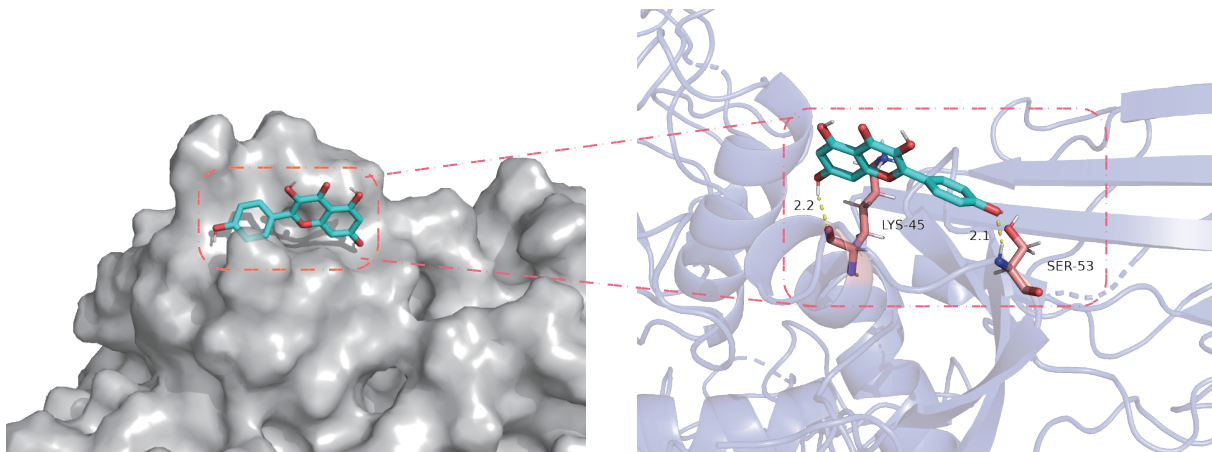

## NOX4-Kaempferol

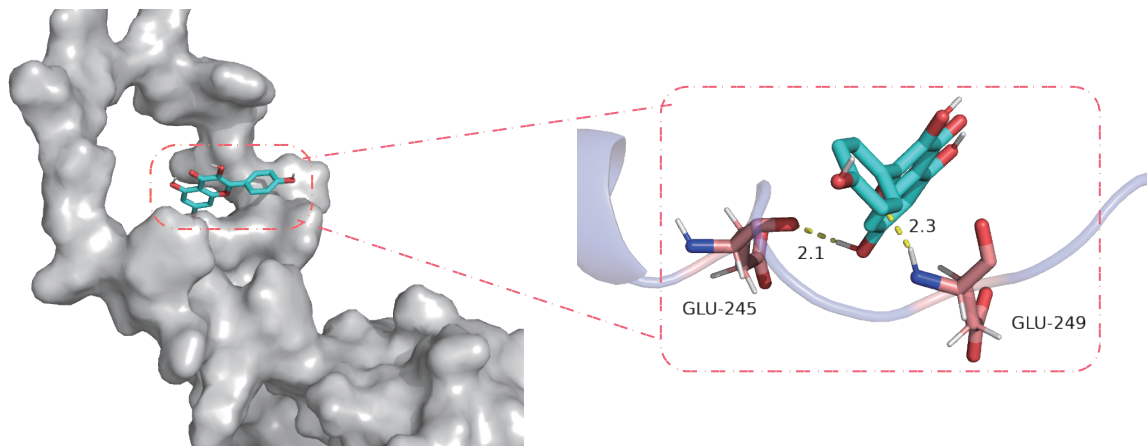

Supplement: Supplementary file 1 [file pharmaceuticals-17-01433-s001.zip › pharmaceuticals-3261423-supplementary/Supplementary File-Figure of molecular docking/COL3A1-Kaempferol+NOX4-Kaempferol.pdf]

## COL3A1-Luteolin

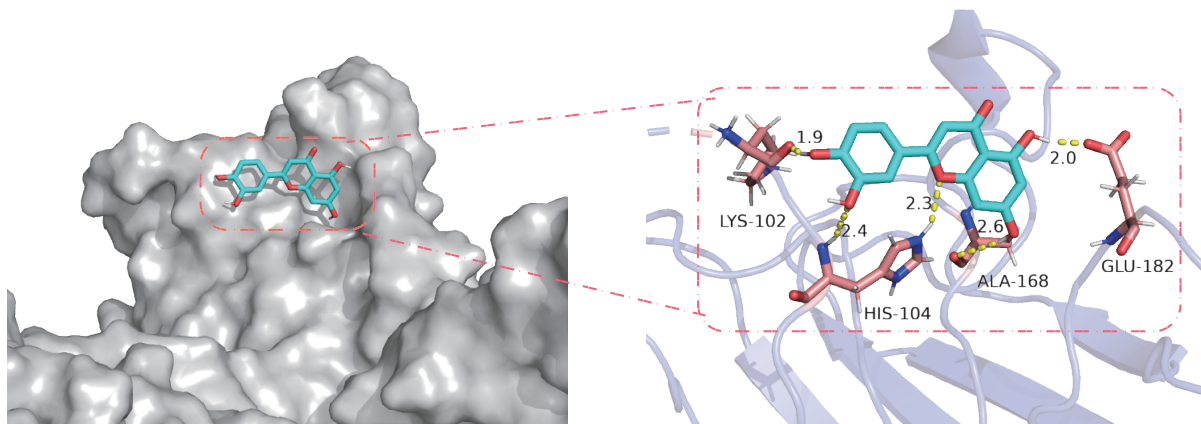

## NOX4-Luteolin

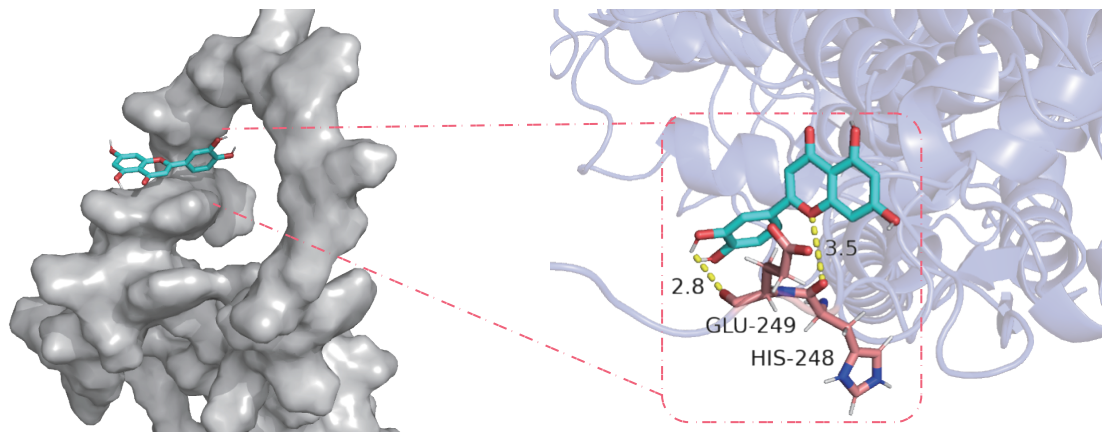

Supplement: Supplementary file 1 [file pharmaceuticals-17-01433-s001.zip › pharmaceuticals-3261423-supplementary/Supplementary File-Figure of molecular docking/COL3A1-Luteolin+NOX4-Luteolin.pdf]

## COL3A1-Naringenin

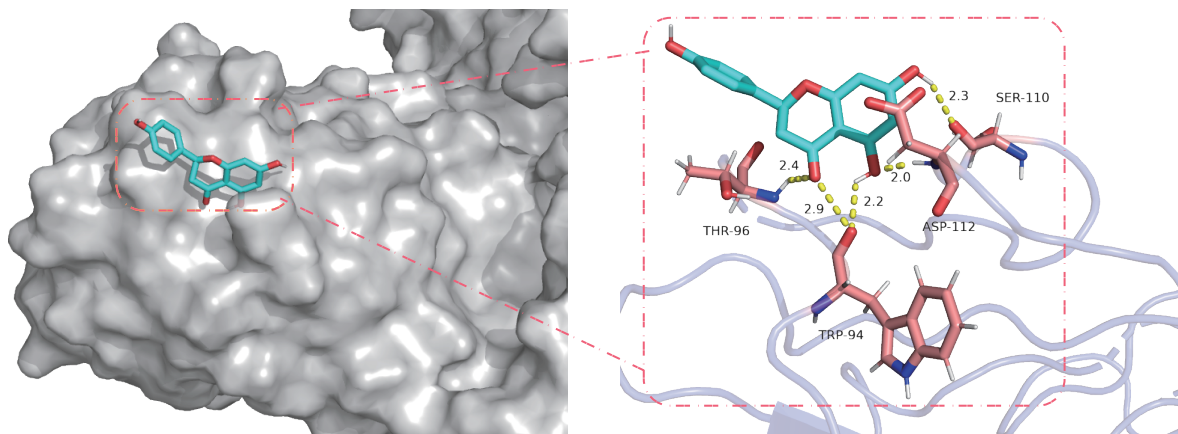

## BGLAP-Salvigenin

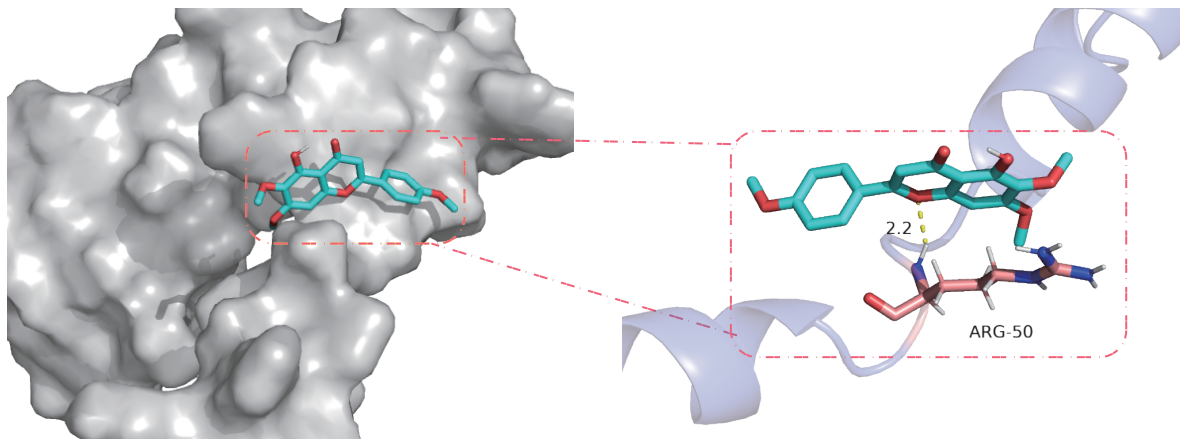

Supplement: Supplementary file 1 [file pharmaceuticals-17-01433-s001.zip › pharmaceuticals-3261423-supplementary/Supplementary File-Figure of molecular docking/COL3A1-Naringenin+BGLAP-Salvigenin.pdf]

## COL3A1-Quercetin

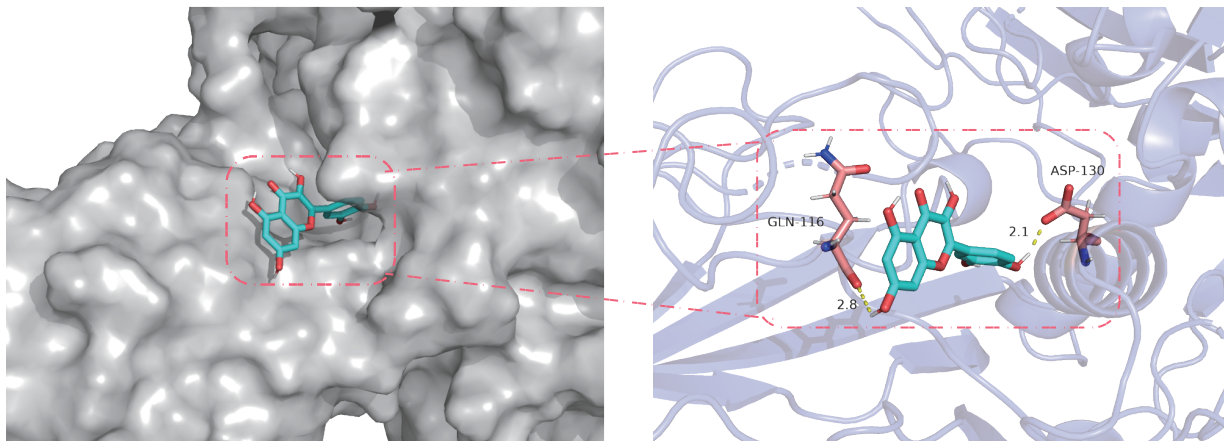

## NOX4-Quercetin

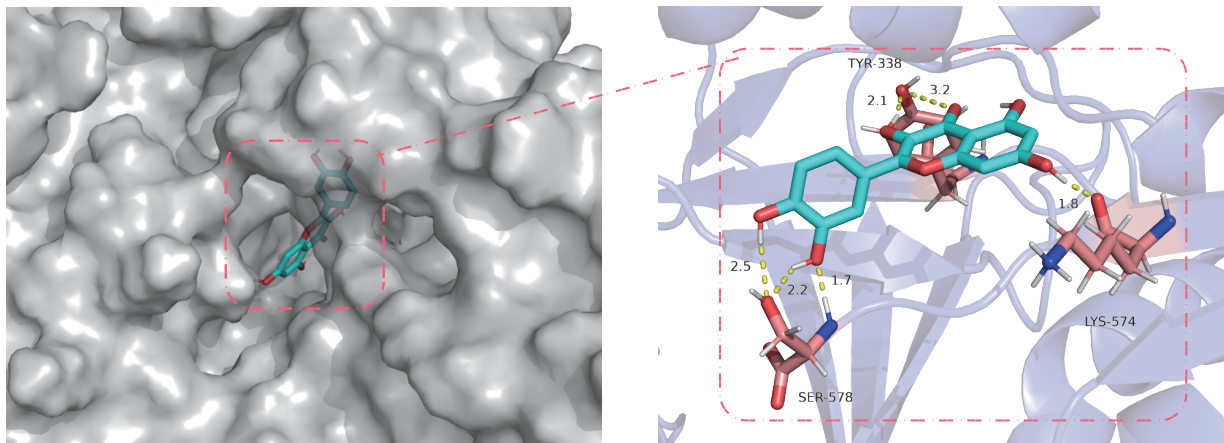

Supplement: Supplementary file 1 [file pharmaceuticals-17-01433-s001.zip › pharmaceuticals-3261423-supplementary/Supplementary File-Figure of molecular docking/COL3A1-Quercetin+NOX4-Quercetin.pdf]
